# YB-1 AP–CSD Forms Cross-β Amyloid Fibrils Without Secondary-Structure Conversion In Vitro

**DOI:** 10.3390/ijms27083553

**Published:** 2026-04-16

**Authors:** Maria A. Timchenko, Oxana V. Galzitskaya, Alexander V. Chulkov, Ilya V. Likhachev, Anna V. Glyakina, Maxim V. Molchanov, Nikolay V. Molochkov, Nikita V. Penkov, Liya G. Bobyleva, Vitalii A. Balobanov, Alexander Ye. Yegorov, Sergey G. Guryanov, Alexey D. Nikulin, Dmitry N. Lyabin, Ivan M. Vikhlyantsev, Alexander G. Bobylev

**Affiliations:** 1 Institute of Theoretical and Experimental Biophysics, Russian Academy of Sciences, Pushchino 142290, Russia; maria_timchenko@mail.ru (M.A.T.); ogalzit@vega.protres.ru (O.V.G.); ivanvikhlyantsev@gmail.com (I.M.V.); 2 Gamaleya Research Center of Epidemiology and Microbiology, Moscow 123098, Russia; 3 Institute of Protein Research, Russian Academy of Sciences, Pushchino 142290, Russiasergei.gurianov@helsinki.fi (S.G.G.);; 4 Department of Fundamental Medicine, Mari State University, Pl. Lenina 1, Yoshkar-Ola 424001, Russia; 5 Institute of Mathematical Problems of Biology, Russian Academy of Sciences, Branch of the Keldysh Institute of Applied Mathematics, Russian Academy of Sciences, Pushchino 142290, Russia; 6 Institute of Cell Biophysics, FRC PSCBR, Russian Academy of Sciences, Pushchino 142290, Russia; nvpenkov@rambler.ru; 7 Scientific Center of Genetics and Life Sciences, Sirius University of Science and Technology, Sirius Federal Territory 354340, Russia

**Keywords:** YB-1, cold shock domain, amyloid fibrils, cross-β structure, protein aggregation, circular dichroism, ATR-FTIR, solution NMR, X-ray fiber diffraction

## Abstract

The central role of YB-1 in messenger ribonucleoprotein particle (mRNP) metabolism and stress-granule biology highlights the importance of defining the determinants of its self-assembly. YB-1 fibrillogenesis has been attributed primarily to the cold shock domain (CSD). Here, we show that the YB-1 fragment spanning residues 1–129 (AP–CSD) form amyloid fibrils under near-physiological ionic strength (0.12–0.15 M KCl). Fibrillization proceeds without a pronounced exponential growth phase and increases approximately linearly over 45–50 h. Far-UV circular dichroism (CD) and attenuated total reflection Fourier-transform infrared spectroscopy (ATR-FTIR) indicate no substantial change in overall secondary-structure content during aggregation. In parallel, ^1^H nuclear magnetic resonance (NMR) spectroscopy reveals the depletion of soluble species, and oriented fiber X-ray diffraction displays the hallmark cross-β reflections at approximately 4.7 Å and 10 Å. The prolonged formation time implies an activation barrier that is unlikely to require global refolding. Instead, it may reflect early association events such as dimerization or other local rearrangements required for primary nucleation, followed by consolidation into stable intermolecular contacts. Aggregation that preserves a largely native-like fold while establishing cross-β order may reduce recognition by cellular quality-control systems that preferentially target globally unfolded or strongly destabilized states. This provides a plausible framework for how YB-1 derived assemblies could persist under stress and during age-associated proteostasis decline.

## 1. Introduction

Y-box-binding proteins (YB proteins) are a family of eukaryotic nucleocytoplasmic proteins that interact with both DNA and RNA. The best-characterized member is Y-box-binding protein 1 (YB-1), initially described as a transcription factor recognizing the Y-box motif in promoters and later as a high-affinity RNA-binding protein [1]. YB-1 carries out a broad spectrum of regulatory functions, including control of transcription, translation, alternative splicing, RNA stability, and DNA repair, as well as participation in cellular stress responses (including genotoxic stress) and in the development of multidrug resistance [2]. Genome-wide analyses have mapped YB-1–RNA interactions and implicated YB-1 in miRNA processing [3]. Moreover, the related YB protein YB-3 can substitute for YB-1 in global mRNA binding [4].

**Figure 1:**
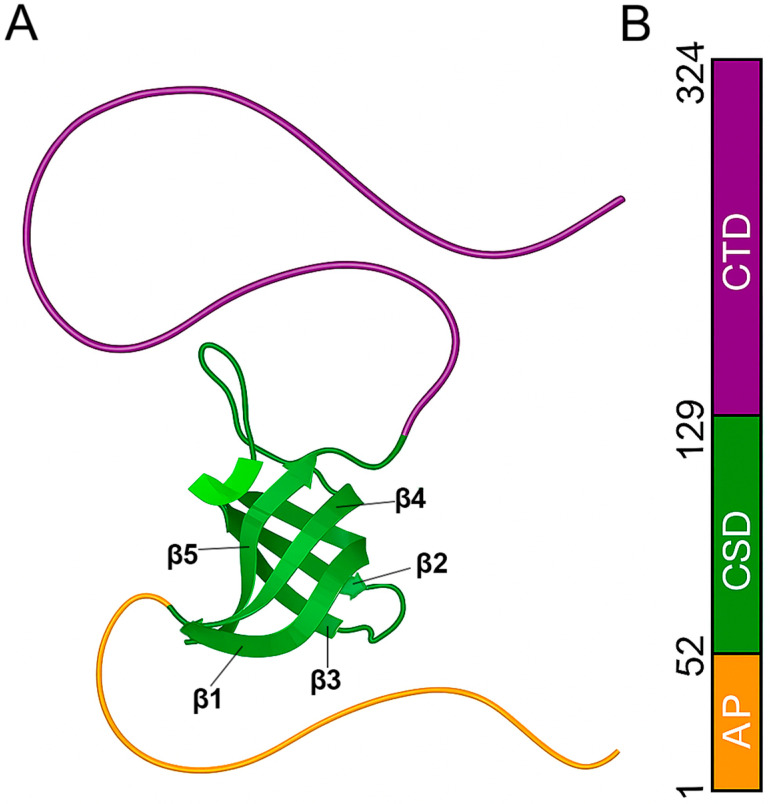
Domain organization of YB-1. (**A**) Crystal structure of the human YB-1 CSD [5]. Residues 88–90, highlighted in light green, adopt short α-helical elements. Tertiary structure of the CSD with schematic N- and C-terminal domains. (**B**) Full-length YB-1 and the truncated constructs used in this study; colored segments indicate AP, CSD, and CTD (domain boundaries as in [5]). The CSD comprises five antiparallel β-strands forming a compact β-barrel. The central β-strands (β2–β3) harbor the RNA-binding motifs RNP1 and RNP2 [11], which are characteristic of CSDs and are also found in RNA-recognition motifs (RRMs) of RNA-binding proteins [12]. Aromatic side chains within these motifs (e.g., Trp65, Phe74, Phe85, His87) mediate π–π stacking with nucleic acids and are critical for the RNA-binding and RNA-unwinding activities of CSD proteins [13].

Structurally, YB-1 comprises three domains: an N-terminal alanine/proline-rich segment (AP), a CSD, and a C-terminal domain (CTD) with alternating charged regions (Figure 1). The central CSD adopts a conserved β-barrel fold and contains the ribonucleoprotein motifs RNP1 and RNP2 motifs. RNA binding is mediated, in part, by π–π stacking of aromatic residues Trp65, Phe74, Phe85, and His87 [5]. The flanking domains (AP and CTD) are predicted and experimentally indicated to be intrinsically disordered. The CTD is thought to augment RNA binding and to contribute to protein multimerization [1]. Despite intensive research, the full-length structure of YB-1 remains unresolved; to date, only the CSD structure has been determined by NMR spectroscopy [6]. The CSD exhibits comparatively low stability: at 25 °C, it retains native conformation in only ~70%, and the thermal transition to the denatured state occurs at ~35 °C, markedly lower than for the *E. coli* cold-shock protein CspA (~56 °C) [7,8,9]. These differences may relate to the elongated loop characteristic of eukaryotic CSDs [10].

Recent studies have shown that YB-1 forms amyloid-like fibrils at high ionic strength (2 M LiCl), displaying hallmarks of amyloid binding to thioflavin T and Congo red and a characteristic X-ray fiber diffraction pattern [14,15]. Electron and atomic force microscopy implicated the CSD as the main fibril-forming module [15]. Electron microscopy images of YB-1/AP–CSD amyloid-like fibrils have been previously reported [15]. In the present study, we used AFM as an independent morphology readout under our aggregation conditions. The isolated CSD has been reported to exhibit limited stability and pronounced conformational dynamics, implying that it can transiently sample partially unfolded conformations under non-denaturing conditions [16]. In the full-length protein, transient intramolecular contacts involving the intrinsically disordered CTD are expected to reduce the accessibility of self-association-competent surfaces on the CSD, including edge β-strands and adjacent loops. Accordingly, truncation of the CTD is expected to increase solvent exposure of these interaction patches and facilitate early association steps such as dimerization or small oligomer formation without invoking extensive global unfolding of the CSD core [16]. The AP can further modulate initiation by increasing the probability of productive intermolecular encounters and providing additional weak contacts, consistent with prior observations that terminal regions modulate YB-1 fibrillization [15]. More broadly, amyloidogenesis is strongly influenced by solution conditions and can be affected by transient heterotypic interactions with other proteins or nucleic acids [17,18,19,20]. In contrast to many amyloid systems that require harsh conditions (extreme pH, temperature, or denaturants) to surmount high activation barriers [17,18,19,20], AP–CSD can aggregate under mild conditions and can do so without major changes in its overall secondary-structure content.

This makes AP–CSD a promising model system for dissecting amyloidogenesis mechanisms and, given the critical role of YB-1 in stress-granule biology, a potentially relevant target in the context of age-related proteostasis decline [21,22,23,24]. Understanding the aggregation of such proteins without extensive structural rearrangement may illuminate mechanisms underlying age-dependent loss of cellular resilience to aggregates.

Importantly, prior work has reported that the AP–CSD fragment (YB-1 residues 1–129) can form amyloid-like fibrils under near-physiological ionic strength (0.15 M KCl) over a timescale of several days (≈92 h) [15]. However, the accompanying structural changes and the extent to which fibrillization proceeds from a native-like CSD fold versus requiring substantial conformational conversion have not been resolved. In particular, direct spectroscopic evidence addressing global secondary-structure conversion during AP–CSD assembly under these mild conditions has been limited. Accordingly, we aimed to characterize the kinetics and structural signatures of AP–CSD fibrillization under near-physiological ionic strength using complementary solution and solid-state readouts, including DLS/AFM, CD/ATR-FTIR, ^1^H NMR, and oriented fiber X-ray diffraction.

## 2. Results

### 2.1. Aggregation of the AP–CSD Fragment

Aggregation of the AP–CSD fragment was monitored by nuclear magnetic resonance (NMR), circular dichroism (CD), dynamic light scattering (DLS), and atomic force microscopy (AFM). Experiments were performed in a near-physiological phosphate buffer containing 120 mM KCl, 5 mM K_2_HPO_4_, and 5 mM KH_2_PO_4_ (pH 7.4). The total observation period was 82 h. To enable parallel structural readouts, the sample was split into two equal portions: one was incubated inside the NMR spectrometer; the other, outside the instrument to collect aliquots for CD measurements.

### 2.2. DLS Analysis

**Figure 2:**
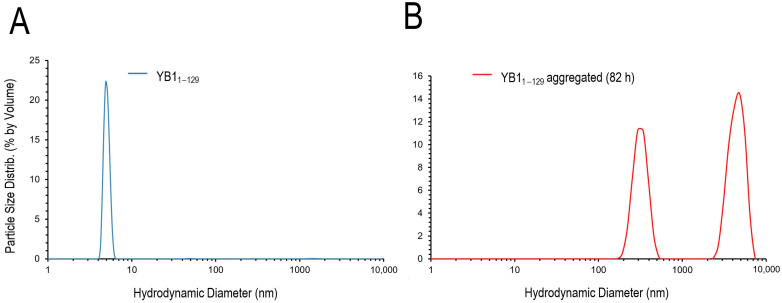
DLS analysis of AP–CSD and its aggregates. Distribution of AP–CSD particles (monomers) (**A**) and AP–CSD aggregates (82 h) (**B**).

At *t* = 0 h, the DLS profile displayed a single predominant peak corresponding to monomeric AP–CSD with a hydrodynamic radius *R*_h_ ≈ 3 nm (Figure 2A). After 82 h of incubation, two size populations were observed in the intensity-weighted distribution: one with *R*_h_ ~ 150 nm contributing to ~62.8% of the total intensity; and the other, with *R*_h_ ~ 2700 nm (~37.2%), consistent with the formation of large aggregates (Figure 2B).

### 2.3. AFM Analysis

**Figure 3:**
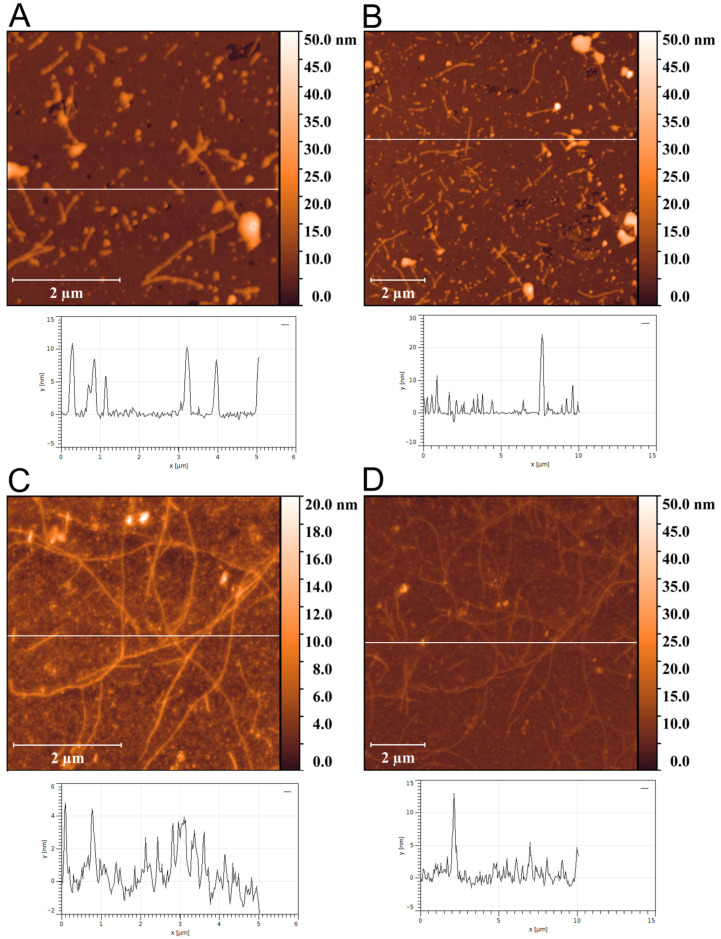
Atomic force microscopy (AFM) of the AP–CSD aggregates. AFM height images of AP–CSD aggregates formed after 12 h (**A**,**B**) and 82 h (**C**,**D**) incubation in aggregation buffer (120 mM KCl, 5 mM K_2_HPO_4_, 5 mM KH_2_PO_4_, pH 7.4) at 24 °C under quiescent conditions (no shaking). Scan areas were 5 × 5 µm and 10 × 10 µm. Scale bars: 2 µm. Purified AP–CSD retained RNA-binding activity as shown by electrophoretic mobility shift assay (EMSA)/native polyacrylamide gel electrophoresis (PAGE) (Supplementary Figure S1B).

AFM was used to visualize the aggregate morphology. Up to 10 h in phosphate buffer (120 mM KCl, 5 mM K_2_HPO_4_, 5 mM KH_2_PO_4_, pH 7.4), no large particles were detected (Supplementary Figure S2). After 12 h of aggregation, we observed oligomers and protofibrils with lengths of ~0.5–1 µm and heights of ~7–10 nm (Figure 3A,B). After 82 h, thin filamentous structures consistent with mature fibrils predominated; fibril heights were ~5–6 nm with lengths > 5 µm (Figure 3C,D). In addition to mature fibrils, a minor population of oligomers and protofibrils persisted.

### 2.4. ^1^H NMR Spectroscopy

**Figure 4:**
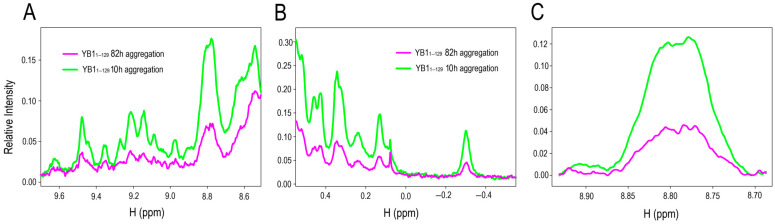
(**A**–**C**) ^1^H NMR spectra of AP–CSD before (green; 10 h) and after (magenta; 82 h) incubation in physiological buffer (120 mM KCl, 5 mM K_2_HPO_4_, 5 mM KH_2_PO_4_; pH 7.4), demonstrating progressive signal attenuation upon aggregation. (**C**) Enlarged view of the 8.70–8.85 ppm region.

To probe changes in molecular mobility and packing during AP–CSD aggregation, ^1^H NMR spectra were recorded after 10 h and 82 h of incubation in a near-physiological buffer (120 mM KCl, 5 mM K_2_HPO_4_, 5 mM KH_2_PO_4_, pH 7.4). The spectra (Figure 4A–C) showed a marked attenuation of signal intensity at 82 h, while peak positions remained largely unchanged. Intensity decreases were evident in both the aliphatic region (0.5–3.0 ppm) and the aromatic/amide region (8.5–9.6 ppm), consistent with the involvement of side chains and the polypeptide backbone in aggregation. The effect was most pronounced at 8.7–8.9 ppm (Figure 4C), characteristic of backbone amide protons, where the signal dropped by more than twofold. Such attenuation reflects reduced molecular mobility and the formation of high-molecular-weight species whose lines broaden beyond NMR detection.

Quantitative analysis of the integrated ^1^H signal intensity revealed the time course of depletion of the NMR-visible soluble pool. During the first 10 h of acquisition (~20 h of aggregation), the relative intensity remained nearly constant (~90–100% of the initial value), consistent with a nucleation period (Supplementary Figure S3). Starting at ~20 h of acquisition (~30 h of aggregation), the intensity decreased progressively, consistent with the growth phase. By 65–70 h of acquisition (~75–80 h of aggregation), the relative intensity declined to ~40%, indicating substantial loss of soluble, fast-relaxing protein.

### 2.5. Secondary Structure Analysis

**Figure 5:**
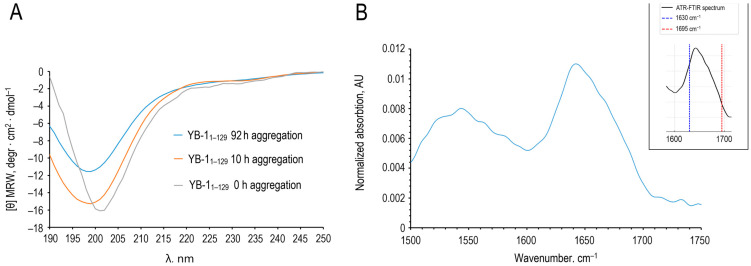
(**A**) Far-UV CD spectra of AP–CSD (YB-1(1–129)) at 0 h (gray), 10 h (orange) and 82 h (blue) of incubation under aggregation conditions (120 mM KCl, 5 mM K_2_HPO_4_, 5 mM KH_2_PO_4_, pH 7.4). (**B**) ATR-FTIR spectrum of AP–CSD fibrils obtained after 82 h under the same conditions. The inset marks the bands at ~1630 and ~1695 cm^−1^ used to calculate the *I*_1695_/*I*_1630_ ratio according to Ruysschaert & Raussens [25], indicating a substantial antiparallel β-sheet contribution.

Far-UV CD spectra collected at multiple time points revealed no appreciable increase in β-content over 82 h of incubation (Figure 5A). The antiparallel β fraction remained at ≈31–34%, and the CD-derived α-helical contribution was minimal, and the disordered component (coils/loops) persisted at ≈47–52% (Table 1). Minor differences in band positions and amplitudes across the far-UV CD spectra were observed as aggregation progressed. Such variations can arise from baseline correction and scaling and can be amplified by increased light scattering from larger assemblies in the far-UV range. Importantly, CD deconvolution yielded comparable secondary-structure fractions across time points (Table 1). ATR-FTIR acquired after 82 h corroborated these observations (Figure 5B), indicating ~36% β-structure and ~52% disordered content. Together, these data argue against a global secondary-structure conversion typical of many amyloids.

Following the procedure of Ruysschaert and Raussens [25], we evaluated the amide I band ratio *I*_1695_/*I*_1630_. For AP–CSD aggregates (82 h), *I*_1695_/*I*_1630_ was ~0.44, which is indicative of antiparallel β-sheet organization (values > 0.25), whereas markedly lower values have been reported for predominantly parallel cross-β fibrils [25]. This is consistent with the presence of a residual population of oligomeric/protofibrillar species even at 82 h, in agreement with the DLS (Figure 2B) and AFM (Figure 3C,D). The amide II region showed a decrease near ~1530 cm^−1^, which can be associated with changes in backbone amide environment and increased ordering. Although the ~1530 cm^−1^ band did not fully vanish, its attenuation was compatible with increased structural ordering, in line with FTIR features discussed by Ruysschaert & Raussens [25].

CD and ATR-FTIR data indicate that AP–CSD aggregates without appreciable changes in overall secondary structure. The persistence of oligomeric species or early intermediates suggests an aggregation pathway that does not require overcoming a large activation barrier, in contrast to many classical amyloid-forming proteins.

### 2.6. Molecular Dynamics and Bioinformatics

**Figure 6:**
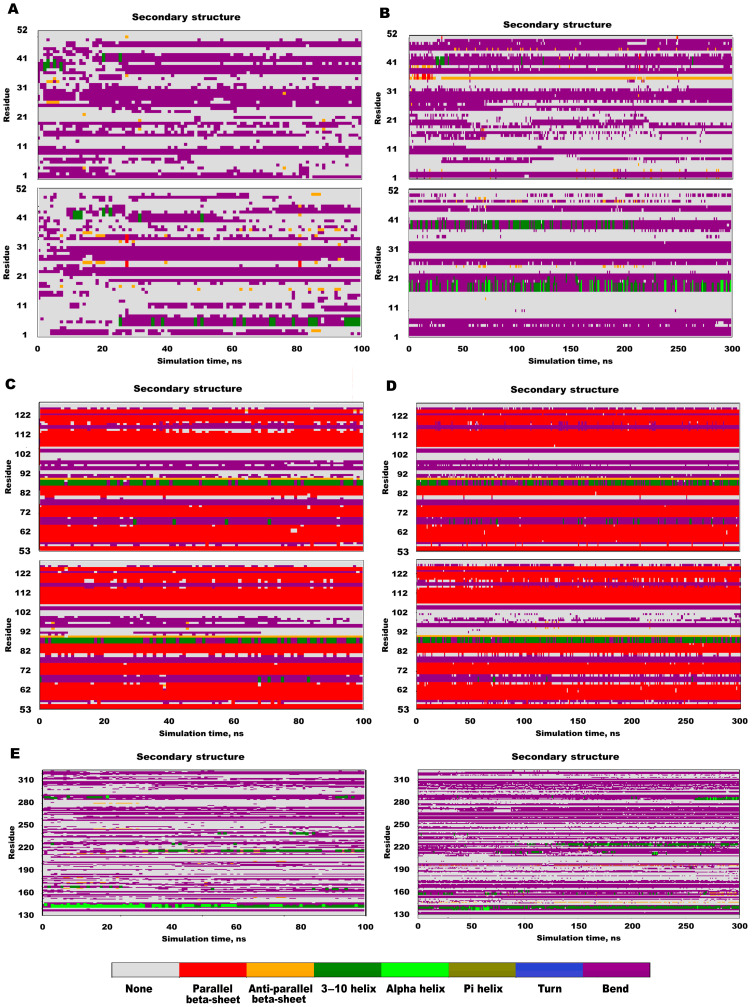
Secondary-structure time courses (DSSP – Define Secondary Structure of Proteins). (**A**,**B**) Full-length YB-1 over 100 and 300 ns; (**C**,**D**) AP–CSD over 100 and 300 ns; (**E**) CTD segment (130–324) extracted for visualization. The CSD β-core (~55–120) persists, whereas AP (~1–40) and CTD are predominantly disordered/turn-rich. Color code: parallel β (red), antiparallel β (purple), α/3-10/π helices (green scale), turns/bends (yellow/brown), none (gray).

All-atom molecular dynamics (MD) simulations of full-length YB-1 (1–324) and the AP–CSD fragment (1–129) suggest an aggregation pathway that preserves the native-like CSD β-barrel and does not involve detectable global refolding within the simulated timescales. Across independent trajectories, the CSD β-barrel remained structurally stable (≈55–120), whereas the AP and the CTD were predominantly flexible/disordered (Figure 6).

Global MD stability metrics support this interpretation: backbone RMSD reached a plateau after early equilibration for full-length YB-1 and remained stable for AP–CSD, whereas residue-level root-mean-square fluctuation (RMSF) was minimal within the CSD β-barrel and maximal in the AP and CTD regions (Supplementary Figure S4).

**Figure 7:**
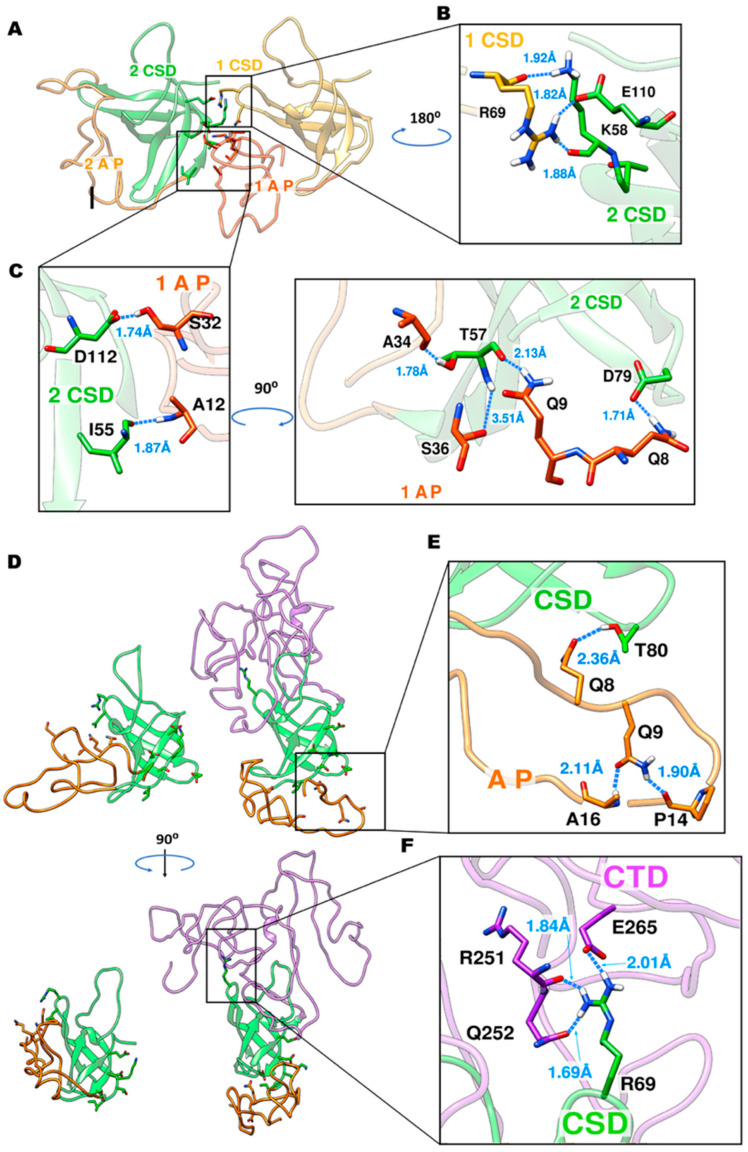
Intermolecular interfaces and residue-level contacts. (**A**–**C**) Two-chain views highlighting CSD–AP and CSD–CSD contacts (H-bonds/salt bridges with distances). (**D**–**F**) Full-length context with CTD; inset shows CTD–CSD interactions (e.g., R251/E265/Q252 with R69) that can modulate interface exposure. These interfaces rationalize strong MM-GBSA ΔGbind (−45…−58 kcal mol^−1^) for AP–CSD oligomers.

To probe self-assembly directly, we simulated a 3×AP–CSD system initiated from randomized orientations. The monomers rapidly formed a persistent intermolecular interface without evidence of global monomer rearrangement, consistent with association driven by native-like edge/β-competent segments rather than secondary-structure conversion (Figure 6). Interfacial analysis identified specific CSD–AP and CSD–CSD contacts (hydrogen-bond and salt-bridge networks) that can plausibly seed oligomerization; importantly, in the full-length context, CTD↔CSD contacts were also observed and may modulate accessibility of the same interaction surface (Figure 7).

Intermolecular energetics further support stable association: MM-GBSA estimates over trajectory ensembles yielded ΔG bind ≈ −45 to −58 kcal·mol^−1^ across three independent 3×AP–CSD simulations, indicating thermodynamically favorable self-assembly under near-physiological ionic strength (Figure 7).

As supporting controls, conformational clustering confirmed that the dominant states were reproducible across replicates (Supplementary Figure S4), and an independent MD-based estimate of dimer interaction energies (PUMA-CUDA) yielded a comparable negative interaction-energy range (Supplementary Figure S5).

**Figure 8:**
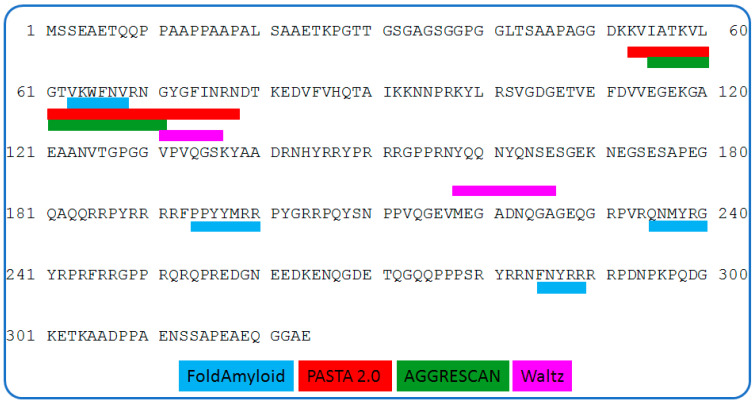
Amyloidogenic regions for the full-length YB-1 protein predicted by the FoldAmyloid, PASTA 2.0, AGGRESCAN, and Waltz programs.

To localize aggregation-prone segments, we predicted amyloidogenic/aggregation-prone regions using four independent algorithms (FoldAmyloid [26], PASTA 2.0 [27], AGGRESCAN [28], and WALTZ [29]), which converged on an aggregation-prone region (APR) spanning residues 53–78 (Figure 8). Extended APR maps across oligomeric states are provided in Supplementary Figure S7.

To provide an independent qualitative estimate of interprotein interaction energetics, we simulated the AP–CSD dimer with PUMA-CUDA (5 ns NPT at 300 K, 1 atm, followed by NVT relaxation at 300 K) and monitored the instantaneous protein–protein interaction energy (not a binding free energy) (Supplementary Figure S6).

Monomers, dimers, and higher-order oligomers of AP–CSD were generated with AlphaFold 3 via the AlphaFold Server (alphafoldserver.com) [30]. In these models, the CSD retains a folded β-barrel architecture across oligomeric assemblies, whereas the AP remains conformationally flexible. This is consistent with a scenario in which self-association can proceed through contacts involving flexible regions without requiring large-scale rearrangement of the CSD core.

The approximate dimensions of the compact oligomeric core in the AlphaFold3 models (~8–10 nm for higher-order oligomers) were qualitatively consistent, at the order-of-magnitude level, with the AFM heights of early aggregates/protofibrils (~7–10 nm), and were compatible with the presence of oligomeric/protofibrillar species alongside mature fibrils in the sample (Supplementary Figure S7 and Supplementary File S1). Importantly, this comparison should be regarded only as a scale-consistency check rather than structural validation: AFM reports the topography of surface-adsorbed and potentially flattened objects (with occasional contributions from crossings/bundles and hydration-dependent effects), whereas AlphaFold3-derived dimensions reflect idealized solution-state models that do not capture polymorphism, surface effects, or heterogeneous packing in fibrillar assemblies.

## 3. Discussion

**Figure 9:**
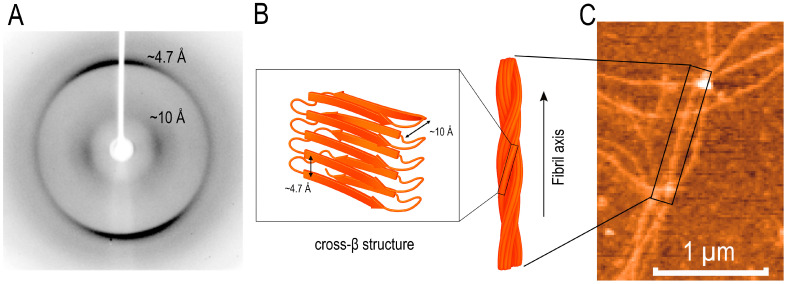
Cross-β architecture of AP–CSD fibrils revealed by oriented-fiber X-ray diffraction. (**A**) Oriented fiber X-ray diffraction pattern of the AP–CSD fibrils (YB-1 residues 1–129) for 82 h in 0.20 M KCl showing the characteristic ~4.7 Å meridional reflection (β-strand spacing along the fibril axis) and ~10 Å equatorial reflection (inter-sheet spacing), consistent with a cross-β arrangement. (**B**) Schematic representation of the cross-β packing within an amyloid fibril illustrating the correspondence between the ~4.7 Å and ~10 Å spacings and the fibril axis (not to scale). (**C**) Representative AFM image of the fibrillar network used for oriented-fiber preparation; the boxed region highlights aligned fibrils. Fibrils were formed after 82 h of AP–CSD incubation.

In this work, we show that the YB-1 fragment comprising the alanine/proline-rich segment and the cold shock domain (AP–CSD; residues 1–129) forms amyloid fibrils under near-physiological conditions (0.12–0.15 M KCl) without appreciable changes in overall secondary-structure content. Concordant CD and ATR-FTIR data indicate comparable β-content and a substantial disordered fraction before and after aggregation, whereas ^1^H NMR reports the conversion of soluble species into NMR-invisible high-molecular-weight assemblies (signal attenuation with minimal chemical-shift changes). Oriented fiber X-ray diffraction of fibrils formed at 0.2 M KCl revealed the hallmark cross-β reflections (~4.7 Å meridional and ~10 Å equatorial) (Figure 9) despite no detectable increase in overall β-content by CD/ATR-FTIR. Together, these findings are consistent with an assembly pathway that preserves the folded CSD β-barrel core.

Our results are consistent with a model in which cross-β order arises primarily through the intermolecular packing of short segments from flexible regions (the AP segment and edge β-strands of the CSD) while the internal CSD β-barrel remains largely intact. MD simulations support this interpretation: DSSP/RMSF analyses indicate a stable β-core (≈55–120) with elevated mobility at the termini, and in a 3×AP–CSD assembly model, persistent interfaces form without detectable global refolding within the simulated timescales. MM-GBSA estimates further suggest thermodynamically favorable association (ΔG_bind ≈ −45 to −58 kcal·mol^−1^), consistent with robust interprotein contacts. Accordingly, AP–CSD self-assembly can be rationalized by intermolecular packing of native-like β-competent/edge segments (APR, residues 53–78), maintaining the overall secondary-structure content (CD/FTIR) while establishing regular intermolecular β-contacts. The resulting inter-strand and inter-sheet spacings are consistent with the cross-β diffraction pattern (~4.7 Å along the fibril axis and ~10–11 Å between β-sheets).

Kinetically, the near-linear decline of the integrated ^1^H NMR signal—which reflects the depletion of the soluble fraction—deviates from the classical nucleation–growth scheme due to the absence of a pronounced lag phase. The most conservative interpretation is a “stretched” primary-nucleation process in which extended contacts gradually form on flexible segments, without necessarily requiring extensive unfolding. Similar aggregation scenarios from native-like or partially structured states have been reported for other proteins; here, this behavior co-occurs with direct evidence for a cross-β architecture under near-physiological conditions.

The time course of the soluble ^1^H NMR signal showed an extended early phase with only minor changes, followed by an approximately gradual decline, which is compatible with a regime of slow, continuous primary nucleation rather than a sharply separated nucleation-then-growth scenario. In such regimes, nuclei can be generated over a broad time window while elongation proceeds in parallel, and the macroscopic “lag” does not necessarily reflect a single rare nucleation event. This interpretation is consistent with kinetic frameworks that treat nucleation as a continuous process and with analyses emphasizing that apparent lag behavior reflects the interplay of multiple microscopic steps. Accordingly, we use “stretched primary nucleation” here to denote distributed, time-extended primary nucleation that can proceed via the gradual formation of productive intermolecular contacts, in line with the minimal secondary-structure changes observed by CD/FTIR [31,32,33].

Comparison with full-length YB-1 underscores the role of the CTD. The intact protein forms fibrils at high ionic strength (2 M LiCl), whereas aggregation is suppressed at 0.12–0.15 M KCl, consistent with an occluding function of the CTD. Removal of the CTD (AP–CSD model) is expected to “unmask” APRs within the AP segment and at the edges of the β-barrel, rendering intermolecular packing favorable already at moderate ionic strength. In the full-length context, CTD↔CSD contacts may reduce the accessibility of these interfaces, acting as a functional “safety-catch” mechanism that raises the threshold for self-assembly.

From a functional perspective, the AP–CSD APR (residues 53–78) maps to the CSD RNA-binding surface (RNP2/RNP1; key residues W65, R69, F74, among others). Upon oligomerization, engagement of this surface in intermolecular contacts would be expected to reduce solvent accessibility of the RNA-binding interface and may weaken RNA chaperone activity (e.g., strand annealing); this inference is based on structural modeling and requires direct functional validation. In the full-length protein, intramolecular CTD↔CSD contacts may compete for this surface and thereby elevate the threshold for self-association. This framework is consistent with competition between RNA binding and self-association and suggests potential regulation by post-translational modifications, limited proteolysis, and features of the cellular milieu (macromolecular crowding; liquid–liquid phase separation, LLPS).

Solution NMR of YB-1C (residues 1–180) under non-aggregating conditions supports a folded CSD core with conformationally dynamic flanking regions and suggests transient sampling of the CSD surface by the disordered tail [10]. Notably, residues perturbed upon nucleic-acid binding map to the same CSD surface that contains the predicted APR (53–78), suggesting a potential overlap between functional RNA-binding and self-association-competent interfaces. However, direct evidence for intermolecular contacts in AP–CSD self-assembly will require oligomer-state NMR or orthogonal approaches such as crosslinking-mass spectrometry.

Biological implications. Given the involvement of YB-1 in stress-granule biology, preservation of a native-like CSD β-core alongside intermolecular packing of flexible β-competent segments (APR/edge β-strands) may facilitate the partial evasion of protein-quality-control systems that predominantly recognize globally unfolded or strongly destabilized states. Stress-dependent modifications (e.g., phosphorylation on flexible regions), limited proteolysis, or dissociation of RNA ligands could increase APR accessibility and lower barriers to self-association without extensive unfolding of the CSD. Future work should evaluate the biological impact of AP–CSD fibrils in cell-based systems. Viability assays such as MTT or related metabolic readouts would help determine whether these assemblies induce measurable cytotoxicity under relevant exposure conditions. These implications follow from structural and modeling observations and will require direct testing in cellular systems. 

## 4. Materials and Methods

### 4.1. Protein Expression and Purification

AP–CSD (residues 1–129) was expressed in *E. coli* and purified essentially as described previously [15]. Briefly, AP–CSD was expressed from the pET22b-YB-1(1–129) plasmid and isolated by ammonium sulfate fractionation followed by chromatography on SP-Sepharose, Phenyl-Sepharose, and MonoS columns (Cytiva, Marlborough, MA, USA; formerly GE Healthcare). The typical yield of purified AP–CSD was ~10 mg·L^−1^ of bacterial culture. Protein concentration was determined by the measurement of *A*_280_ using the calculated molar extinction coefficient for AP–CSD, ε_280_ = 8480 M^−1^·cm^−1^ (ProtParam, ExPASy, SIB, Lausanne, Switzerland; MW = 13.08 kDa). The *A*_280_/*A*_260_ ratio was ~2.0, indicating the absence of detectable nucleic-acid contamination. AP–CSD was stored in 50 mM potassium phosphate buffer (pH 7.4) or in 20 mM HEPES-KOH containing 200 mM KCl (pH 7.4). Protein purity was evaluated by SDS-PAGE (Supplementary Figure S1A). RNA-binding activity of AP–CSD was confirmed by the electrophoretic mobility shift assay (Supplementary Figure S1B).

### 4.2. Aggregation Conditions and Sample Preparation

To assess fibril formation, protein samples (final concentration 30 µM) were incubated (without shaking) at 24 °C for 82 h in aggregation buffer containing 120 mM KCl, 5 mM K_2_HPO_4_, 5 mM KH_2_PO_4_ (pH 7.4). Prior to aggregation, samples stored in 50 mM potassium phosphate buffer were dialyzed (3.5 kDa MWCO (molecular weight cutoff), Thermo FS, Waltham, MA, USA) against the aggregation buffer for 10 h at 4 °C. Subsequently, samples were transferred into the NMR spectrometer for continued incubation (82 h) and monitored in parallel by AFM, CD, and DLS.

### 4.3. X-Ray Diffraction Study

Oriented fiber samples of AP–CSD aggregates were prepared using a simplified stretch-frame protocol as previously described [15,34]. Briefly, an aliquot of the protein gel was transferred into 1 mL of deionized water for 1 min to remove excess salts. The sample was then centrifuged at 12,000 rpm (~13,400× *g*) for 5 min. The resulting pellet was gently transferred and stretched between the waxed ends of two glass capillaries mounted across a Petri dish, and air-dried to produce aligned, rod-like fibers.

X-ray diffraction patterns were recorded using a Microstar generator (Cu Kα radiation, λ = 1.54 Å) with HELIOX optics and a Platinum135 CCD detector (X8 PROTEUM system, Bruker AXS, Madison, WI, USA). The samples were mounted perpendicular to the incident beam and aligned using a 4-axis κ-goniometer (Bruker AXS, Madison, WI, USA). Exposure times ranged from 30 to 60 s depending on signal intensity.

### 4.4. DLS Analysis

DLS experiments were performed to assess the size distribution of AP–CSD aggregates. Protein samples (30 µM) were prepared in a buffer containing 120 mM KCl, 5 mM K_2_HPO_4_, and 5 mM KH_2_PO_4_ (pH 7.4). Measurements were carried out using a Zetasizer Nano ZS instrument (Malvern Instruments Ltd., Malvern, UK).

The autocorrelation functions were collected and processed using the general-purpose analysis algorithm provided by the instrument software Zetasizer Software 7.12 (Malvern, UK). Alternative inversion algorithms yielded comparable size distributions. Light scattering measurements were performed with a laser beam cross-section of ~100 µm. Given the protein concentration, the analyzed scattering volume contained approximately 10^10^ protein molecules per measurement.

For each sample, the correlation function was accumulated over 15 cycles of 15 s each (225 s total acquisition time per run). All reported results represent the mean of three independent experimental replicates.

### 4.5. AFM Imaging

For AFM analysis, the protein solution was diluted to a final concentration of 3 µM. A 5 µL aliquot was deposited onto freshly cleaved mica (specific gravity (water = 1.0): 2.7, chemical formula: K_2_O·Al_2_O_3_·SiO_2_, hardness on Mohs scale of hardness: 2–2.25, thickness = 0.15 mm (0.006″), lateral size = 15 × 15 mm, NT-MDT) and incubated for 15 min in a humid chamber to facilitate adsorption. After incubation, the substrate was gently rinsed twice with ultrapure water and air-dried at room temperature.

AFM imaging was performed using a scanning probe microscope (NTEGRA VITA, NT-MDT, Zelenograd, Russia) in semi-contact (tapping) mode under ambient conditions. Standard silicon cantilevers (NSG03, NT-MDT) were used, with a nominal resonance frequency of 47–150 kHz, a force constant of 0.35–6.06 N/m, and an apex curvature radius of approximately 10 nm.

AFM measurements were performed in three independent aggregation experiments prepared from the same purified AP–CSD batch. These experiments were conducted on different dates, with a >6-month interval between the first and third experiment. Each experiment included independent sample deposition on freshly cleaved mica and independent imaging. The scan size, resolution, and image processing parameters were kept constant across all replicates. Image processing and profile analysis were performed using Nova (NT-MDT) and Gwyddion v2.66 software (http://gwyddion.net, accessed on 17 June 2024).

### 4.6. ^1^H NMR

One-dimensional (1D) ^1^H NMR spectra were recorded at room temperature using a Bruker Avance III 600 MHz spectrometer (Bruker BioSpin, Rheinstetten, Germany) (The Core Facilities Center, Institute of Theoretical and Experimental Biophysics, Russian Academy of Sciences). Standard pulse sequences from the Bruker pulse program library were used.

For NMR experiments, we used an aqueous solution containing 10% deuterium oxide (D_2_O) (99.9%, Chemstore, Saint-Petersburg, Russia). The NMR sample consisted of AP–CSD at a concentration of 0.68 mg/mL in a buffer at pH 7.4 containing 120 mM KCl, 5 mM K_2_HPO_4_, 5 mM KH_2_PO_4_, and 10% D_2_O. Measurements were performed in a Shigemi tube with a sample volume of 250 μL.

Water suppression was achieved using pre-saturation with the ZGPR pulse sequence. Spectra were acquired with 32 to 1024 scans, depending on the signal-to-noise ratio requirements. The relaxation delay between scans was set to 10 s. Free induction decays (FIDs) were collected with 96,000 complex points over an acquisition time of 2.272 s and a spectral width of 24 ppm. The 90° pulse length was calibrated at 11 µs. Prior to Fourier transformation, FIDs were zero-filled to 128,000 points. The spectra were processed in the TOPSPIN program (Bruker, Rheinstetten, Germany).

Chemical shift referencing was performed using the signal of 3-(trimethylsilyl)propionic acid-d_4_ sodium salt (TSP), assigned to 0.00 ppm. Spectra were continuously recorded over a period of 82 h to monitor the kinetics of AP–CSD aggregation in real-time.

### 4.7. CD Spectroscopy

Far-UV CD spectra (190–250 nm) were recorded using a J-810 CD spectropolarimeter (JASCO, Tokyo, Japan) equipped with a 0.1-cm pathlength quartz cuvette and continuously purged with nitrogen gas. Samples contained 30 µM AP–CSD in 120 mM KCl, 5 mM K_2_HPO_4_, and 5 mM KH_2_PO_4_ (pH 7.4). Each sample was measured in triplicate. Raw spectra were processed using Spectra Manager software (Version 2.02.06, JASCO). Three scans were averaged and smoothed. A corresponding buffer spectrum was recorded under identical conditions, averaged, and subtracted to obtain the final CD spectrum of the sample. Protein concentration was calculated from absorbance at 280 nm using the formula:

*C* = *A*_280_/(*l* × ε),

where *A*_280_ is the absorbance at 280 nm, *l* is the optical pathlength in cm, and ε is the molar extinction coefficient (calculated using ExPASy ProtParam).

Molar ellipticity [θ] (deg·cm^2^·dmol^−1^) was calculated using the formula:

[θ]λ = θλ × RMW/(*l* × *c*),

where θλ is the measured ellipticity (in millidegrees), RMW is the mean residue molecular weight (derived from amino acid sequence), *l* is the pathlength in mm, and *c* is protein concentration in mg/mL.

Secondary-structure content was estimated using the CONTIN/LL algorithm from the CDPro software suite (https://www.bmb.colostate.edu/cdpro/ accessed on 7 April 2026) [35]. The mean root square deviation between the experimental and fitted spectra did not exceed 6%.

### 4.8. Attenuated Total Reflectance Fourier Transform Infrared Spectroscopy

ATR-FTIR measurements were performed to assess the secondary structure of AP–CSD fibrils formed after 82 h of aggregation. Infrared spectra were recorded on a Nicolet 6700 FTIR spectrometer (Thermo Fisher Scientific, Waltham, MA, USA) equipped with a deuterated triglycine sulfate detector and a ZnSe ATR crystal.

Aliquots of 1.5 µL of protein solution (6 mg/mL, corresponding to ~459 µM AP–CSD based on MW = 13.08 kDa) were deposited onto the ATR surface and air-dried to form thin protein films. Spectra were collected in the mid-IR region (650–4000 cm^−1^) with 256 co-added scans at a spectral resolution of 4 cm^−1^. Background spectra were collected under identical conditions and automatically subtracted. Spectra were processed using OMNIC V9.2 software (Thermo Fisher Scientific), which included ATR correction (for depth of penetration and refractive index) and baseline correction.

### 4.9. Molecular Dynamics Simulations

Structures of full-length YB-1 (residues 1–324) and AP–CSD were obtained from AlphaFold3 models and prepared in AMBER 24 using the ff19SB protein force field with OPC water. Each system was neutralized, and the ionic strength adjusted to 0.15 M KCl (K^+^/Cl^−^ counterions). After energy minimization, systems were equilibrated in the NVT and NPT ensembles at 303 K and 1 atm using a Langevin thermostat and an isotropic barostat. Production trajectories were propagated with pmemd.cuda (GPU), with a 2 fs time step, SHAKE constraints on bonds to hydrogens, particle-mesh Ewald electrostatics, and snapshot saving every 10 ps. For single-chain YB-1 and AP–CSD, we performed runs of up to 100–300 ns per replicate; for the 3×AP–CSD self-assembly model, we generated three independent 300-ns trajectories from distinct randomized starting orientations. Trajectories were analyzed with cpptraj (backbone RMSD/RMSF, radius of gyration, DSSP secondary structure, and clustering by backbone RMSD). Intermolecular energetics were estimated by molecular mechanics generalized Born surface area (MM-GBSA) over trajectory ensembles, reporting ΔG_bind as the mean over snapshots; three independent simulations yielded −45 to −58 kcal·mol^−1^.

As an auxiliary cross-check, dimer interaction energies were also evaluated with the PUMA-CUDA molecular dynamics package using an AMBER family force field [36] and the TIP3P water model [37]. Simulations employed periodic boundary conditions; the dimer was solvated in a cubic water box extending by approximately two solvent layers beyond the solute. Water molecules sterically overlapping the protein were removed prior to minimization. The resulting system comprised 28,870 atoms in total (3652 protein atoms and 8406 water molecules). Trajectories were analyzed with the Trajectory Analyzer of the Molecular Dynamics Toolkit [38].

## 5. Conclusions

In this study, we show that the YB-1 fragment comprising the alanine/proline-rich segment and the cold shock domain (AP–CSD; residues 1–129) forms amyloid fibrils in 0.12–0.15 M KCl without detectable changes in overall secondary-structure content. CD/ATR-FTIR indicated comparable secondary-structure fractions before and after aggregation, ^1^H NMR reported the conversion of soluble species into high-molecular-weight assemblies, and oriented fiber X-ray diffraction revealed the hallmark cross-β reflections (~4.7 and ~10 Å). Mechanistically, our data are consistent with fibrillogenesis driven by the intermolecular packing of short segments from flexible regions (APR 53–78, the AP segment, and edge β-strands of the CSD) while preserving a near-native CSD β-barrel. Molecular dynamics simulations and MM-GBSA binding free-energy estimates (ΔG_bind ≈ −45 to −58 kcal·mol^−1^) support the formation of stable interfaces; in the full-length protein, the C-terminal domain may act as a safety catch by shielding APRs and raising the threshold for self-association. The predicted overlap of the APR with the CSD RNP surface (RNP1/RNP2) suggests potential competition between RNA binding and self-association; engagement of this surface in APR-mediated contacts is expected to reduce its solvent accessibility and limit nucleic-acid binding. Given the role of YB-1 in stress granules, these findings generate testable predictions for the regulation of aggregation by post-translational modifications, limited proteolysis, and features of the cellular milieu (macromolecular crowding and LLPS). Overall, the work reveals latent amyloidogenic potential of the YB-1 CSD core and proposes a biophysically coherent framework for regulating its self-association, with relevance to RNA-binding proteins with similar domain architectures.

## Figures and Tables

**Table 1:** Secondary structure content in AP–CSD samples determined by the CD and ATR-FTIR methods.

CD	α, %	β, % Antiparallel	β, % Parallel	Turn, %	Other
0 h aggregation	0.4 ± 0.3	34.2 ± 1.7	0.0	17.5 ± 0.9	47.9 ± 2.4
10 h aggregation	2.8 ± 0.3	32.7 ± 1.6	0.0	18.1 ± 0.9	46.4 ± 2.3
82 h aggregation	0.0 ± 0.3	31.9 ± 2.6	0.0	16.4 ± 1.3	51.6 ± 4.1
**ATR-FTIR**	**α, %**	**β, %**	**RC, %**		
82 h aggregation	12 ± 3	36 ± 7	52 ± 8		

**Note:** Uncertainties (±) for CD-derived fractions are approximate and were estimated from the CD fitting residuals (RMSD/nRMSD) using ~5% relative uncertainty for spectra with good fits and ~8% for the spectrum with higher residuals. For very small fractions (≤0.5%), a minimum uncertainty of ±0.3 percentage points was applied to avoid false precision. RC—random coil; Nrmsd—root-mean-square deviation, normalized RMSD.

## Data Availability

The original contributions presented in this study are included in the article/Supplementary Materials. Further inquiries can be directed to the corresponding author.

## Supplementary Materials

The following supporting information can be downloaded at: https://www.mdpi.com/article/10.3390/ijms27083553/s1.

## Abbreviations

FTIR, Fourier transform infrared spectroscopy; DTT, dithiothreitol; SDS-PAGE, sodium dodecyl sulfate-polyacrylamide gel electrophoresis; AFM, atomic force microscopy.
