# Supplementary material for: YB-1 AP–CSD Forms Cross-β Amyloid Fibrils Without Secondary-Structure Conversion In Vitro
Source: Int J Mol Sci. 2026 Apr 16;27(8):3553. doi: 10.3390/ijms27083553 (PMC13116819; doi:10.3390/ijms27083553)
Supplement: Supplementary file 1 [file ijms-27-03553-s001.zip › ijms-4234853-supplementary.pdf]

Supplementary Figures

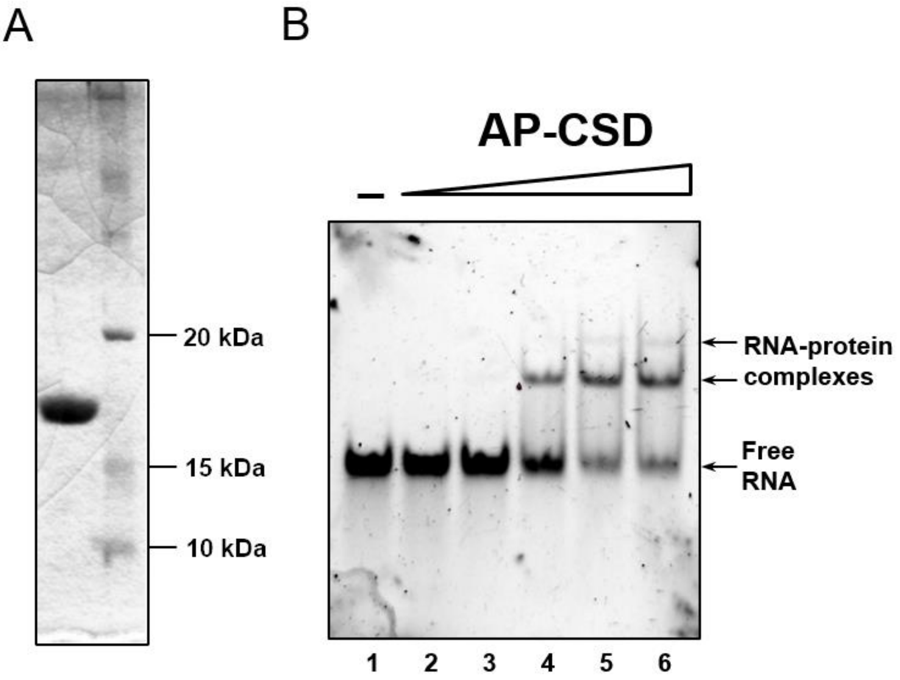

Supplementary Figure S1. (A) SDS–PAGE of purified recombinant YB-1(1–129) (AP–CSD). Left lane: YB-1(1–129) after purification. Right lane: molecular-weight marker (kDa). Coomassie Brilliant Blue staining. The dominant band migrates at the expected apparent molecular mass (~14–15 kDa). (B) RNA-binding activity of recombinant AP–CSD. The FAM-labeled synthetic RNA fragment (30 nucleotides, 0.5 pmol) was incubated in a final volume of 10 μL (10 mM HEPES-KOH [pH 7.6], 150 mM KCl) for 10 min at 30°C with increasing amounts of AP–CSD (6 pmol, lane 2; 12 pmol, lane 3; 24 pmol, lane 4; 48 pmol, lane 5; 96 pmol, lane 6). RNA-protein complexes were analyzed by PAGE under nondenaturing conditions and visualized on a ChemiDoc MP Imaging System (Bio-Rad).

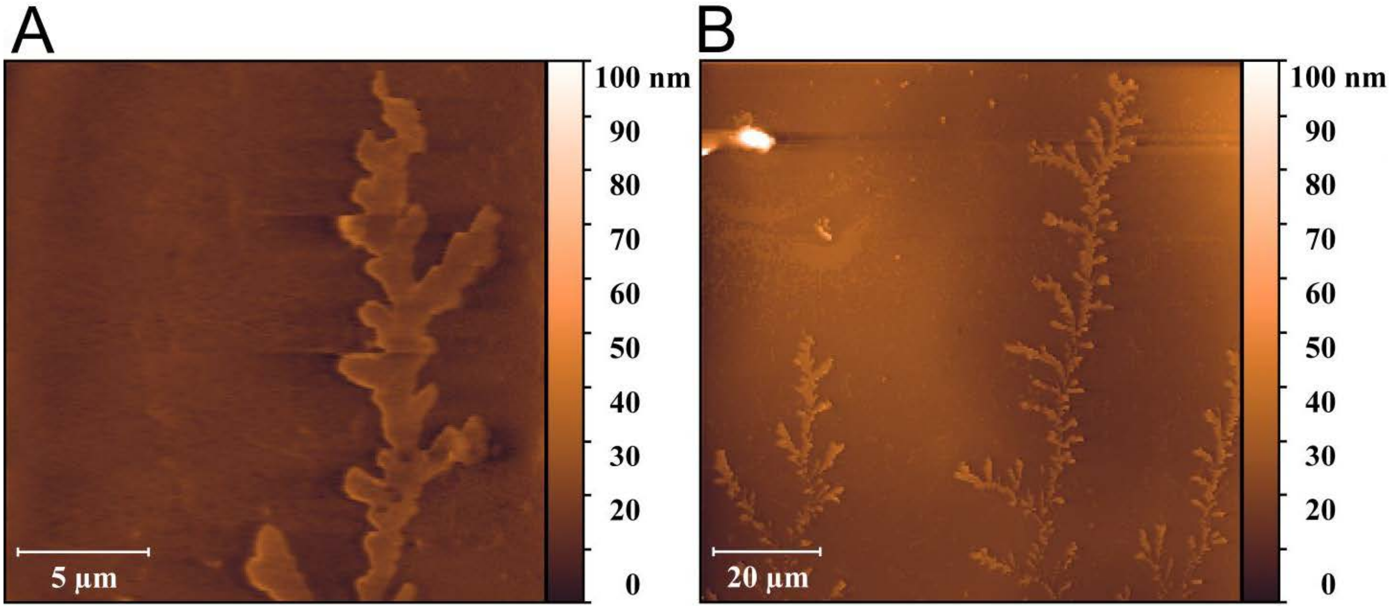

Supplementary Figure S2. Atomic force microscopy of AP–CSD. Time course of aggregate formation: 10 h under the following conditions: 120 mM KCl, 5 mM K_2HPO_4, and 5 mM KH_2PO_4 (pH 7.4) at 4°C. Salt-deposition-like structures are observed. Tree-like features observed in the 10-h control are likely attributable to salt/buffer deposition during drying and are interpreted as background rather than protein aggregates.

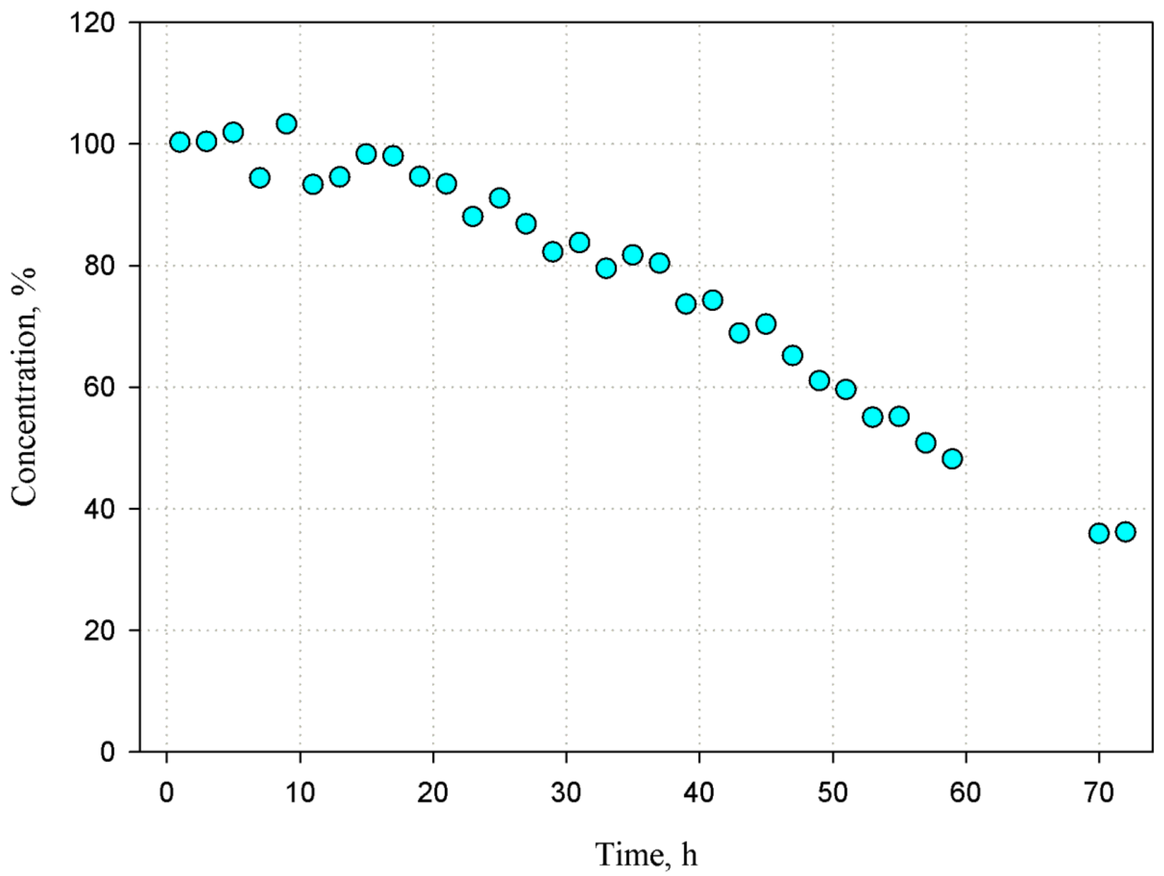

Supplementary Figure S3. YB-1 concentration decrease, NMR data.

The plot shows the decrease in the soluble AP–CSD pool over time, quantified as the normalized total ^{1}H NMR signal intensity (integral) relative to the first spectrum. Prior to NMR monitoring, the sample was dialyzed for 10 h at 4°C against aggregation buffer (120 mM KCl, 5 mM K_2HPO_4, 5 mM KH_2PO4, pH 7.4). After dialysis, the sample was transferred to the NMR spectrometer and spectra were recorded continuously for 72 h to follow aggregation in real time.

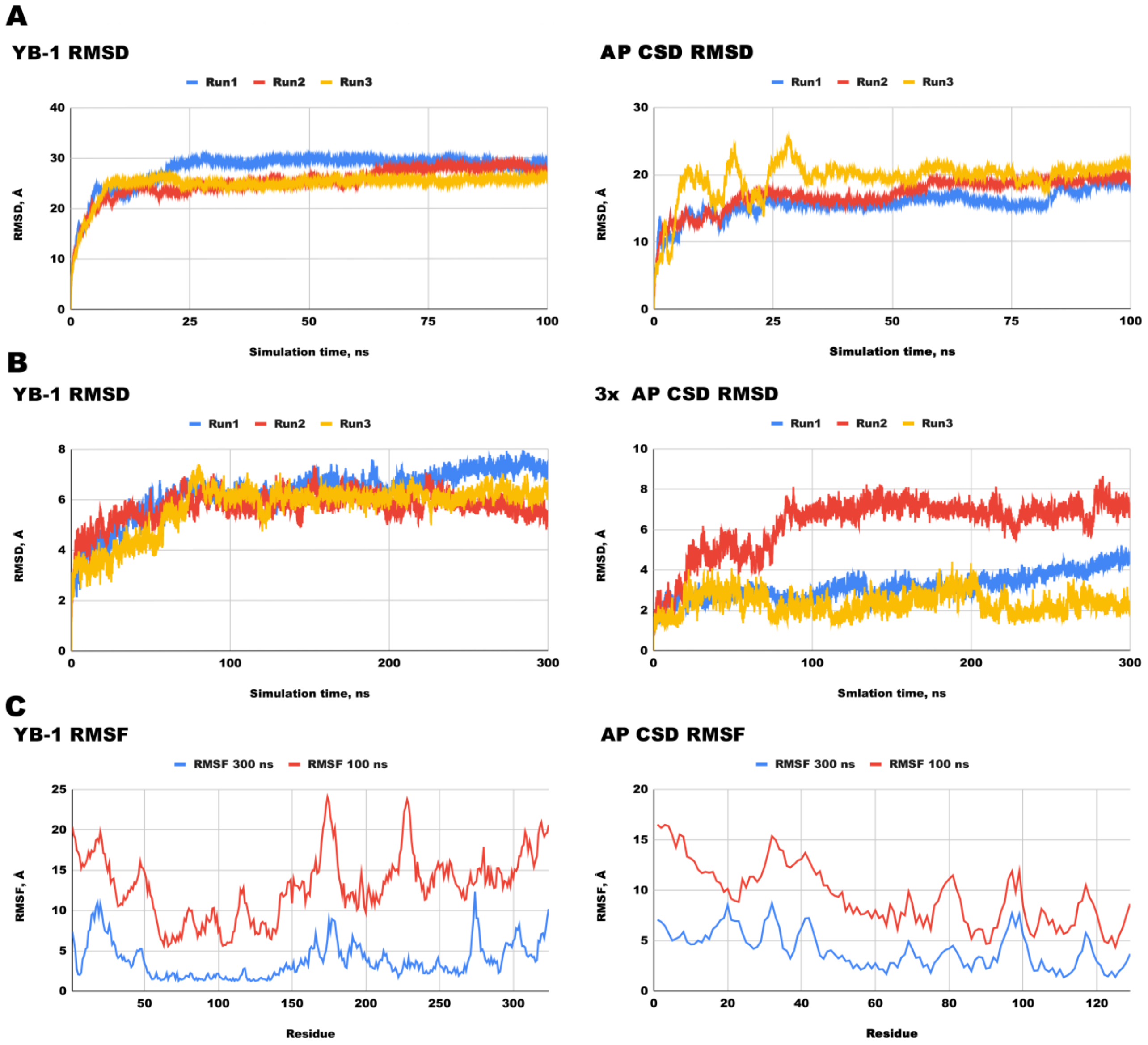

Supplementary Figure S4. Global stability and flexibility (RMSD/RMSF). (A) Early RMSD (0–100 ns) for YB-1 and AP–CSD. (B) Long trajectories (up to 300 ns) for YB-1 (left) and 3×AP–CSD (right); the trimer shows rapid stabilization and low RMSD spread. (C) RMSF per residue: minima at the CSD β-barrel; maxima at AP and CTD.

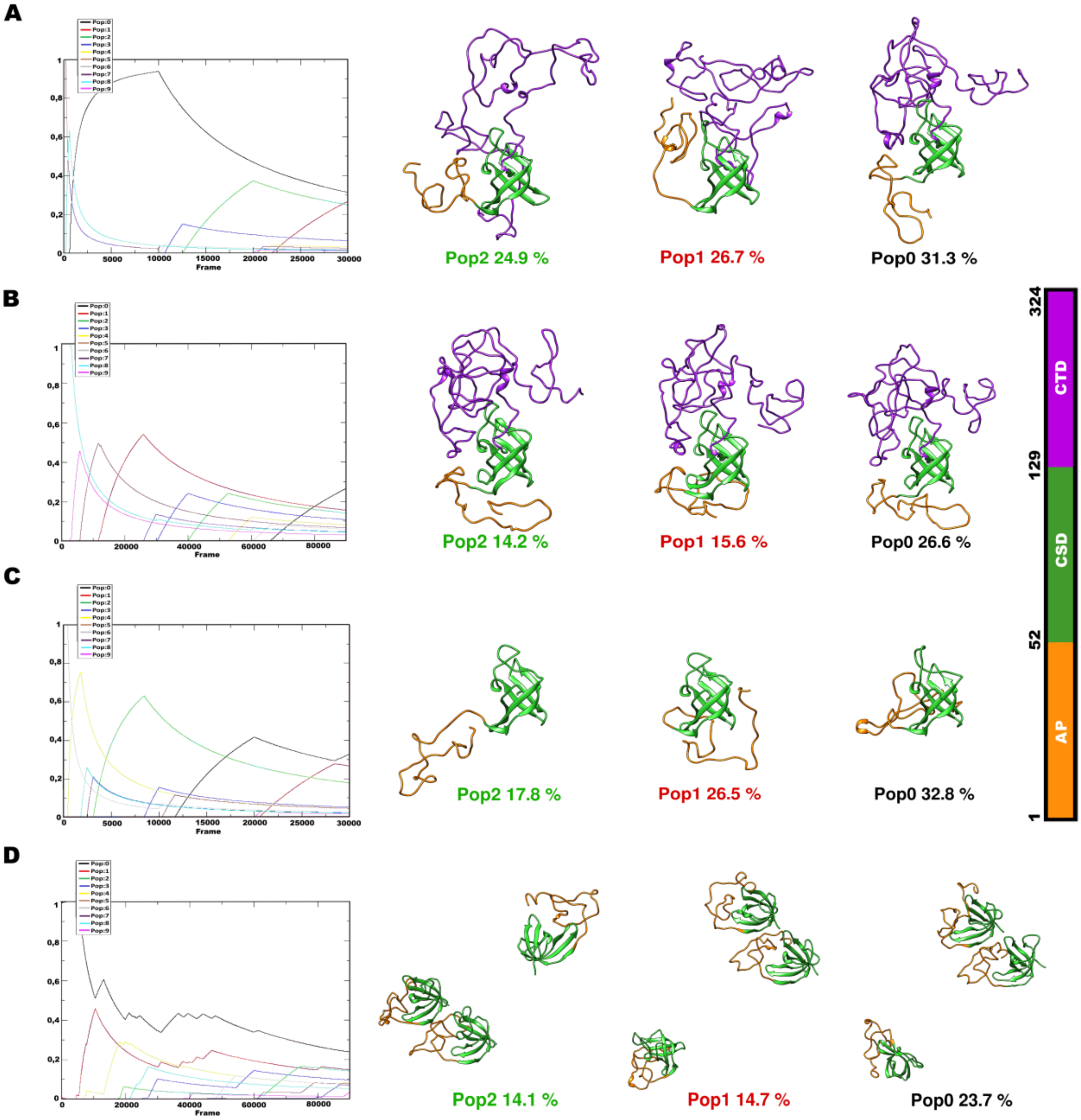

Supplementary Figure S5. Conformational clustering. Representative centroids of the three dominant clusters (Pop0/Pop1/Pop2 ~15–33%) for YB-1 (top) and AP–CSD (bottom). All clusters preserve the β-barrel while reorienting flexible termini, consistent with aggregation via exposed disordered patches rather than refolding.

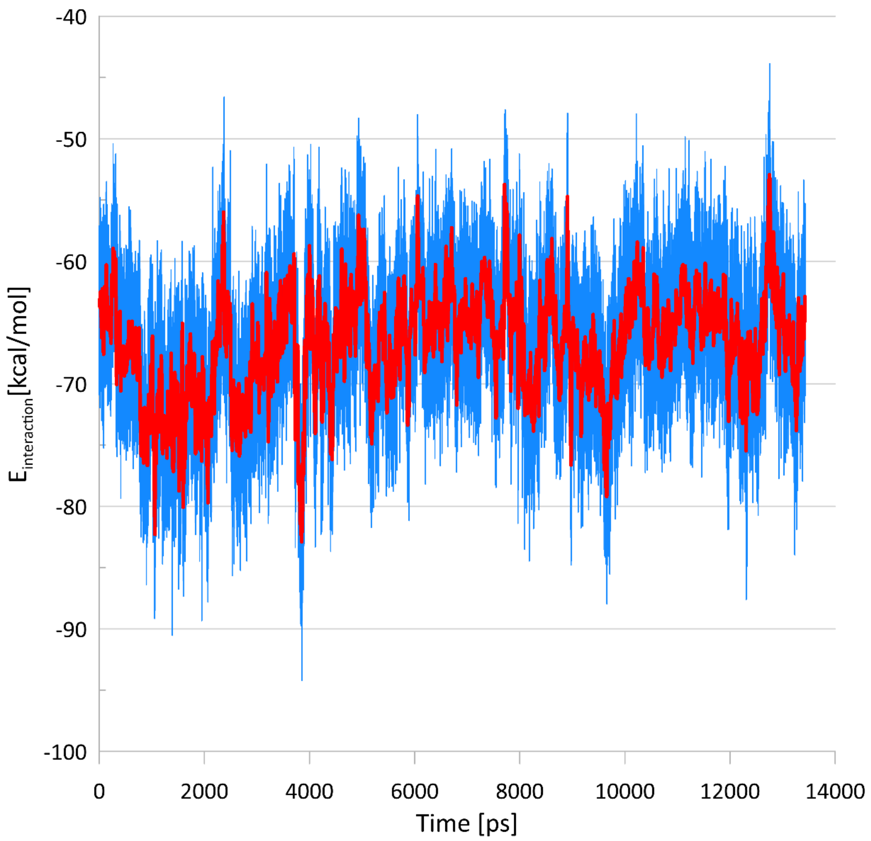

Supplementary Figure S6. Interaction energy between the two protein monomers: blue curve, instantaneous values sampled every 0.1 ps; red curve, values averaged over 10 ps.

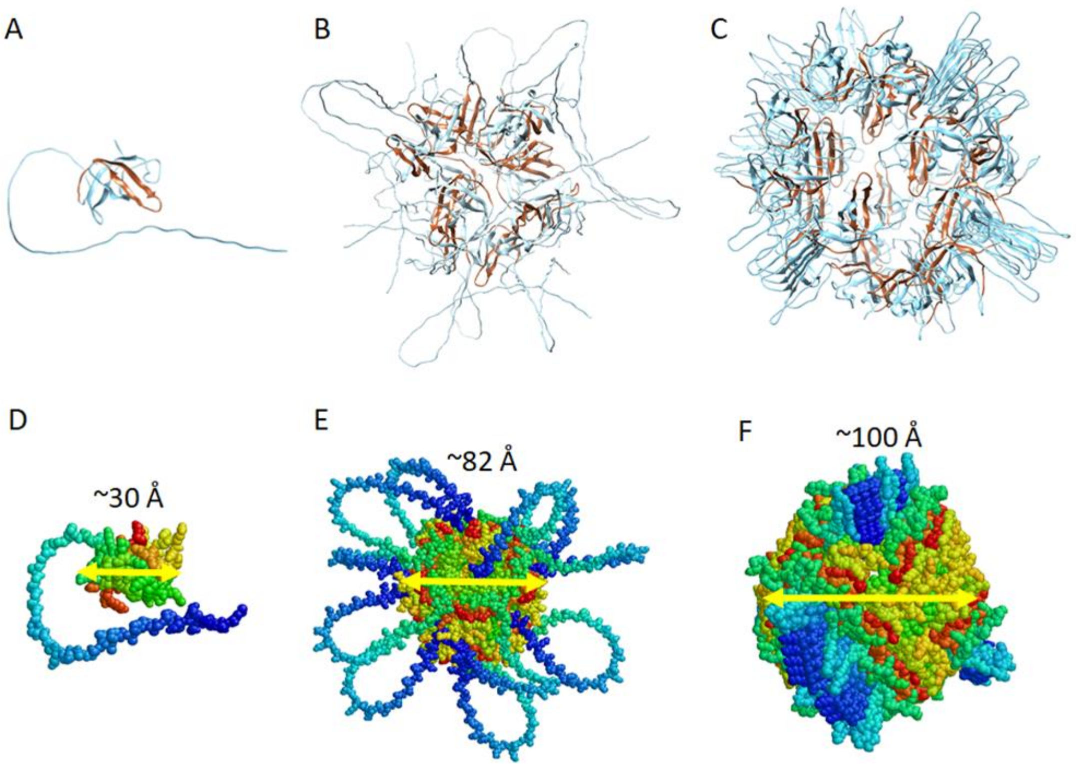

Supplementary Figure S7**.** The predicted amyloidogenic region as a union of four programs (residues 53–78) is colored brown for the monomer (A), the dodecamer (B), and the 24-mer (C) for AP–CSD. Sizes of the globular part for monomer (D), dodecamer (E), and 24-mer (F) are indicated.
