# Supplementary figures and images for: YB-1 AP–CSD Forms Cross-β Amyloid Fibrils Without Secondary-Structure Conversion In Vitro
Source: Int J Mol Sci. 2026 Apr 16;27(8):3553. doi: 10.3390/ijms27083553 (PMC13116819; doi:10.3390/ijms27083553)

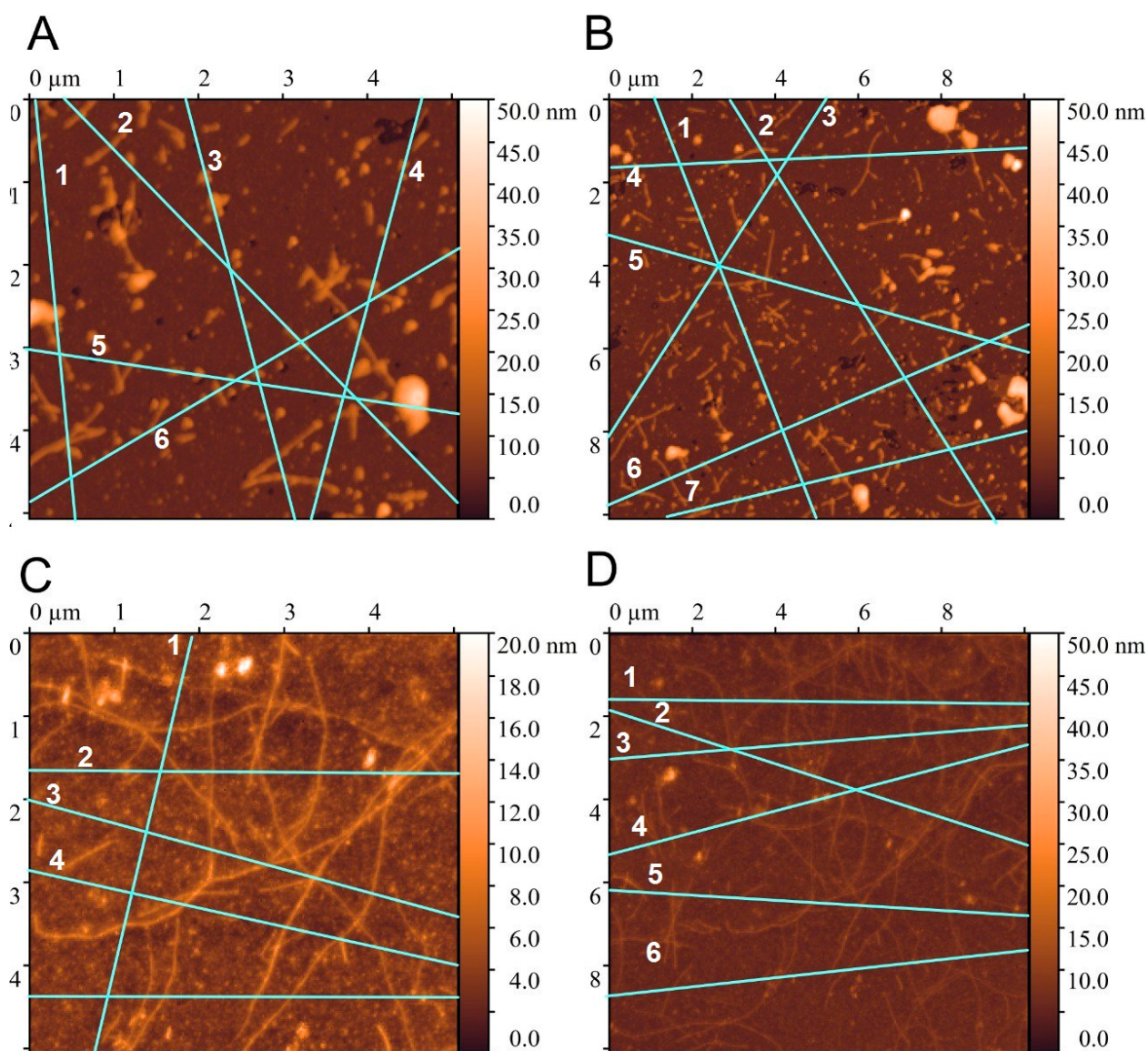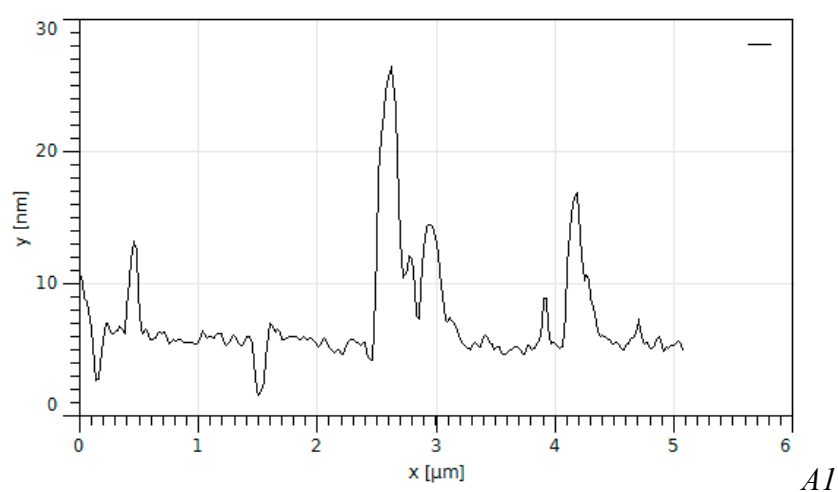

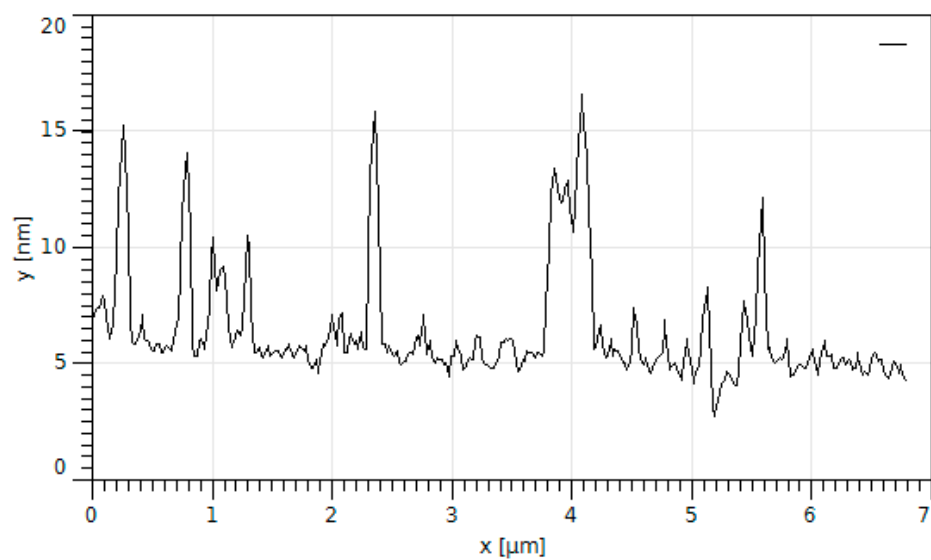

A2

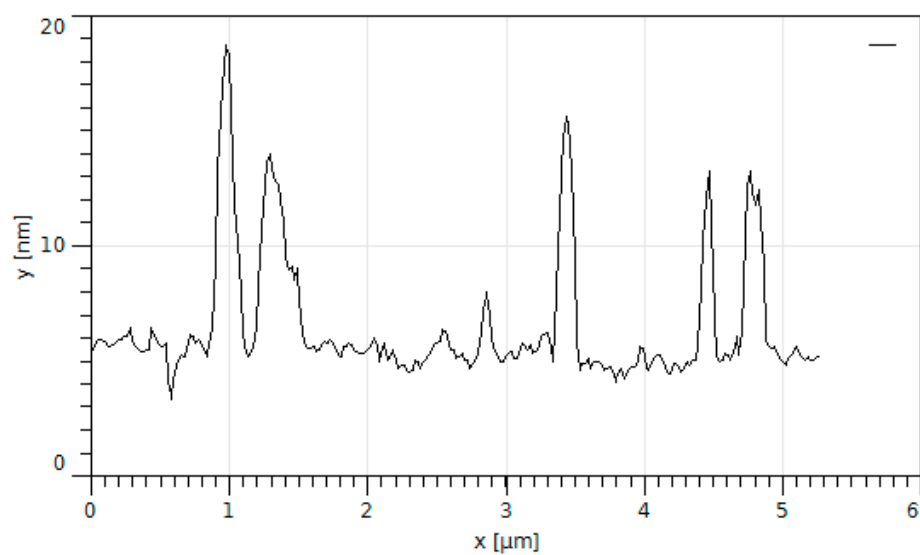

A3

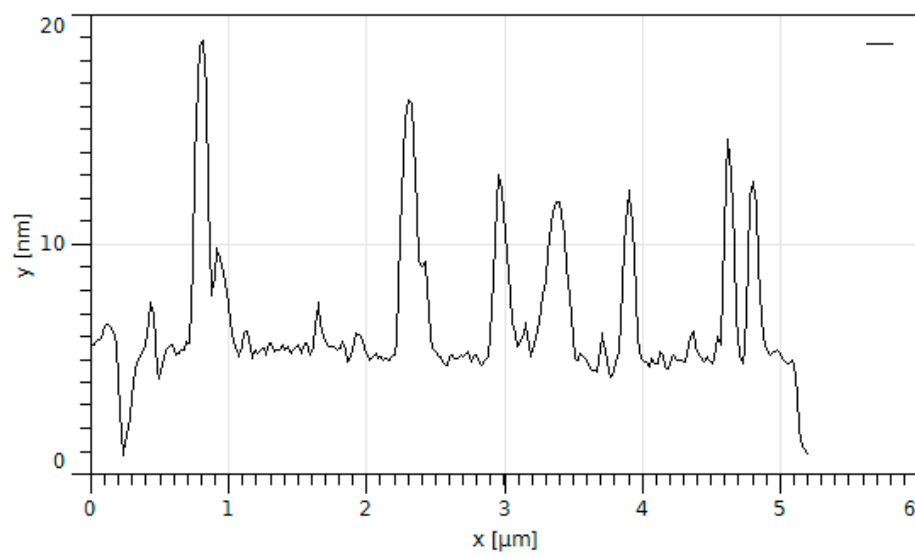

A4

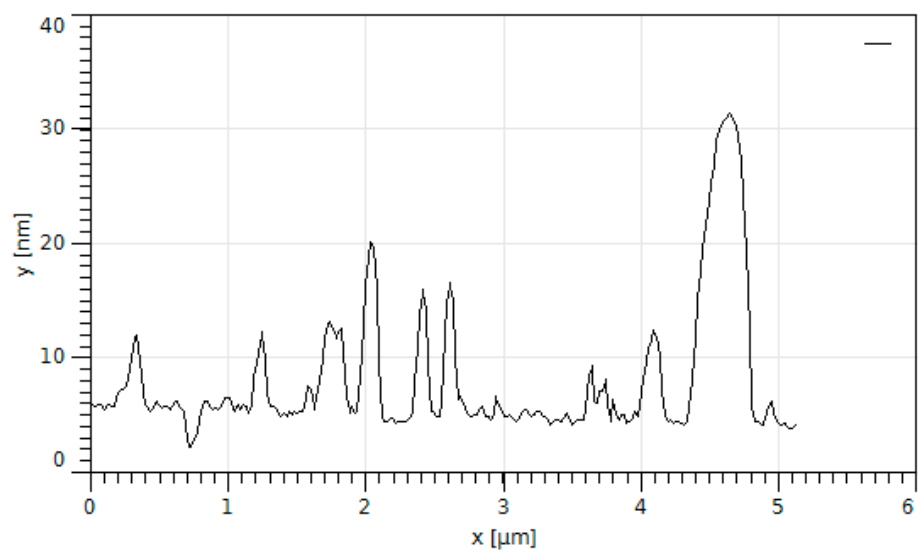

A5

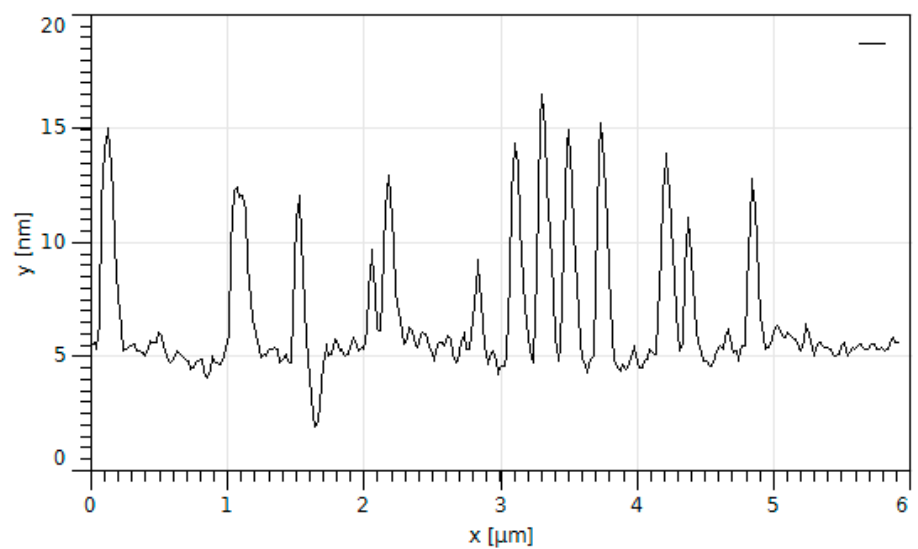

A6

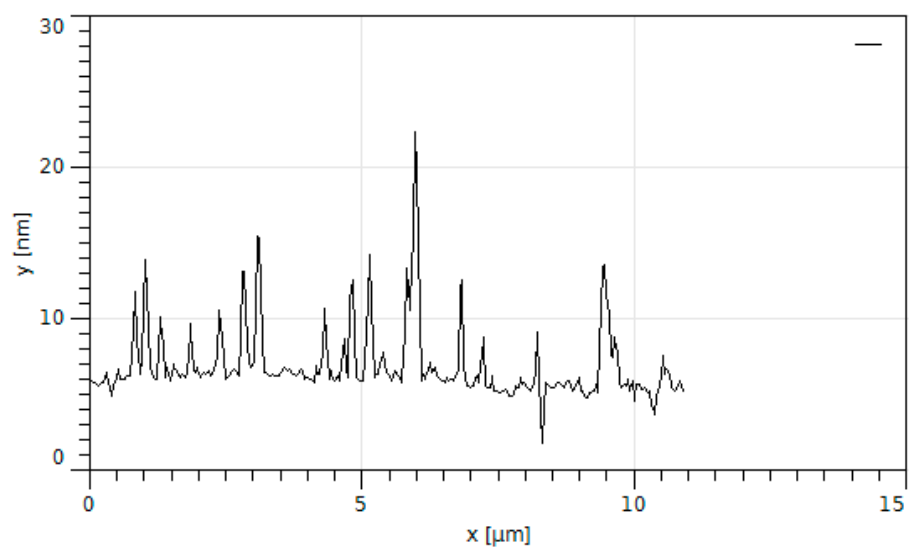

B1

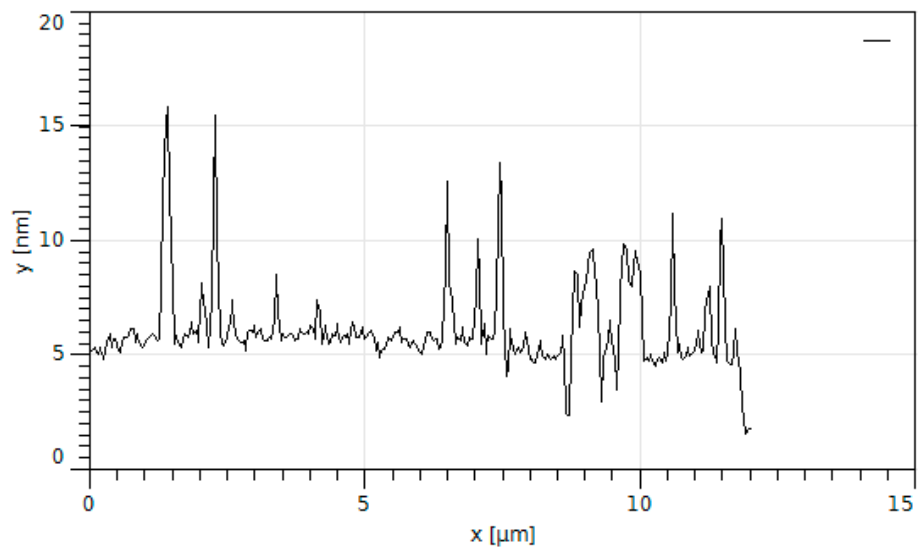

*B2*

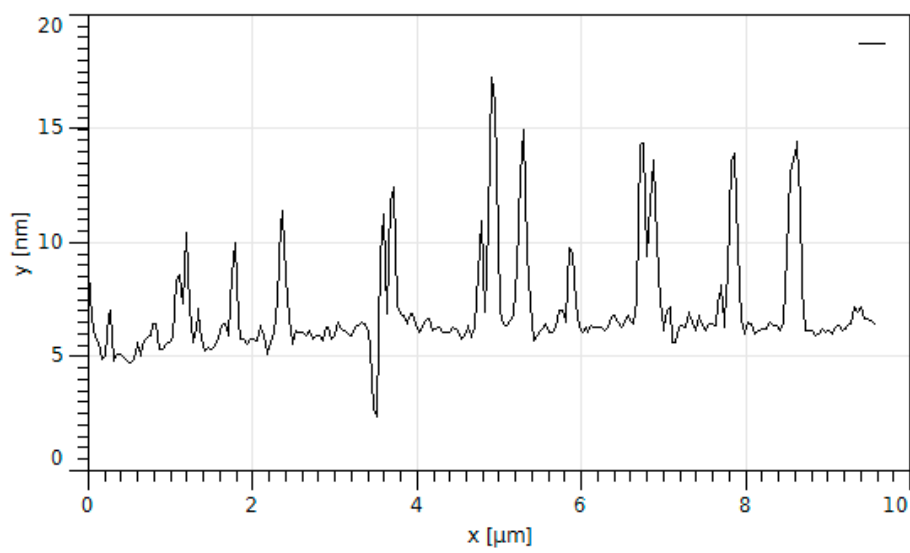

*B3*

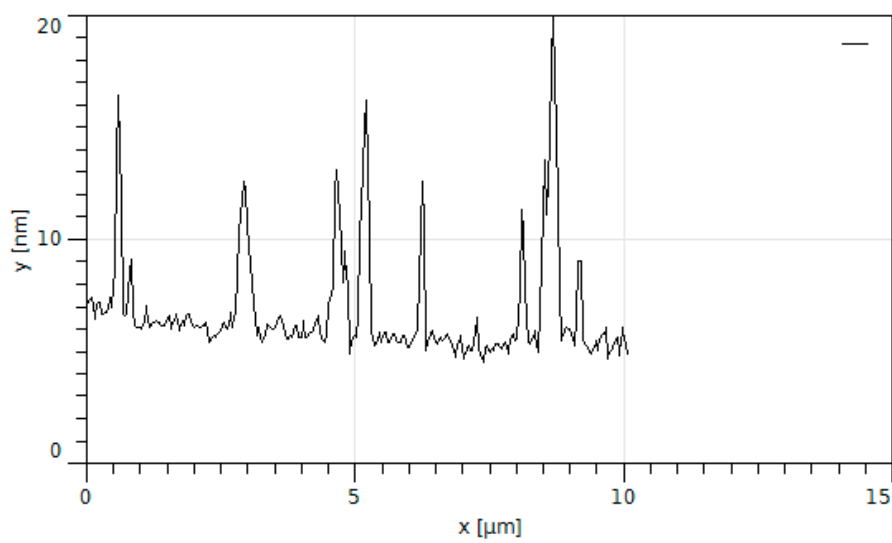

*B4*

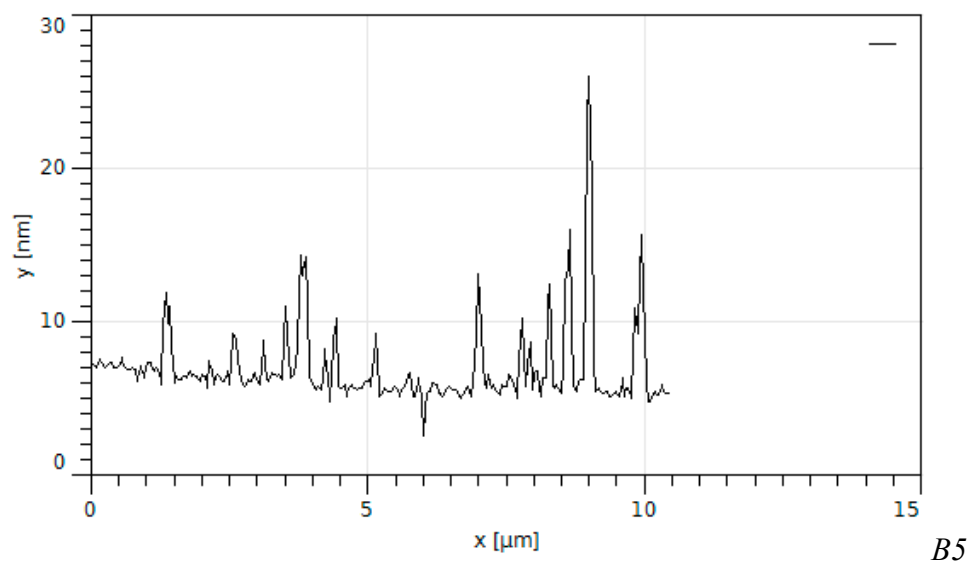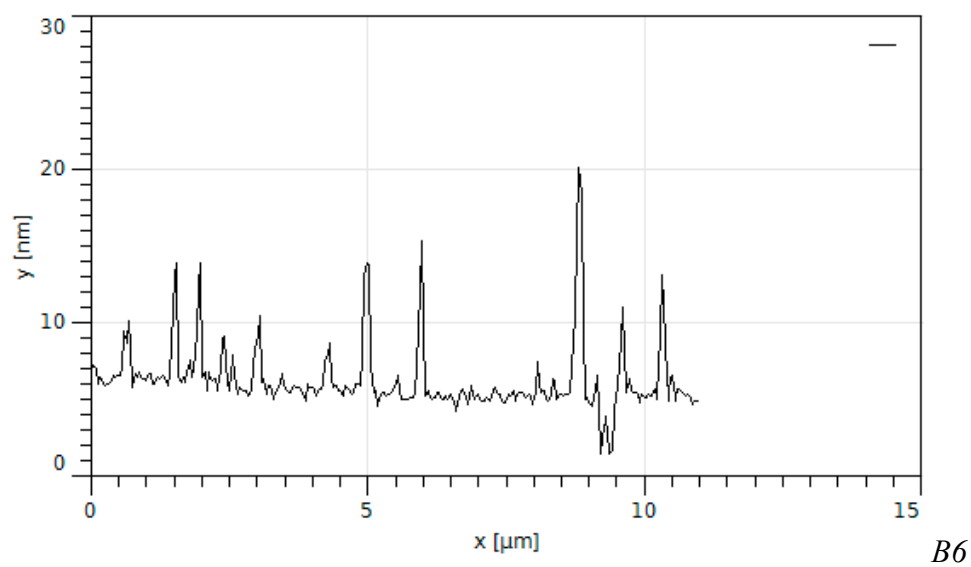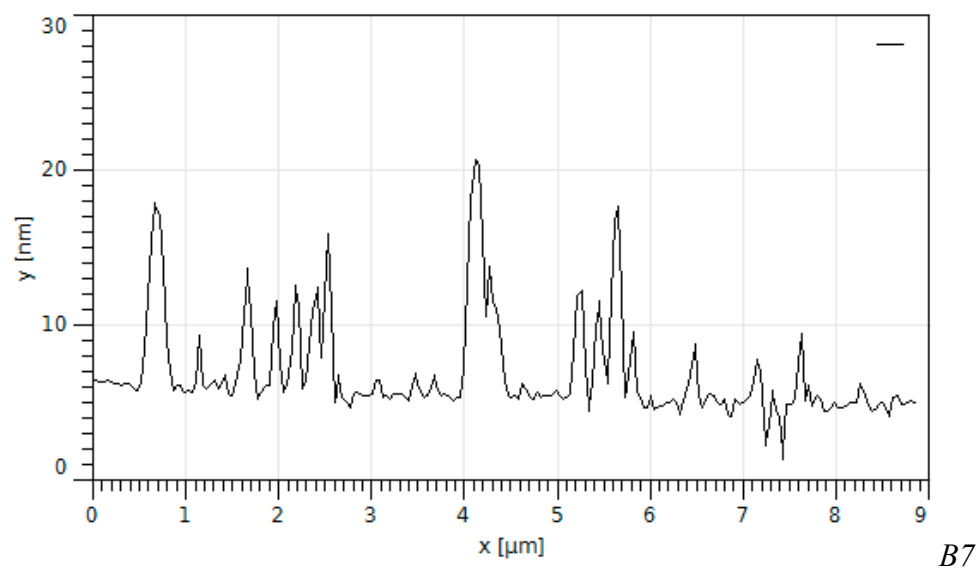

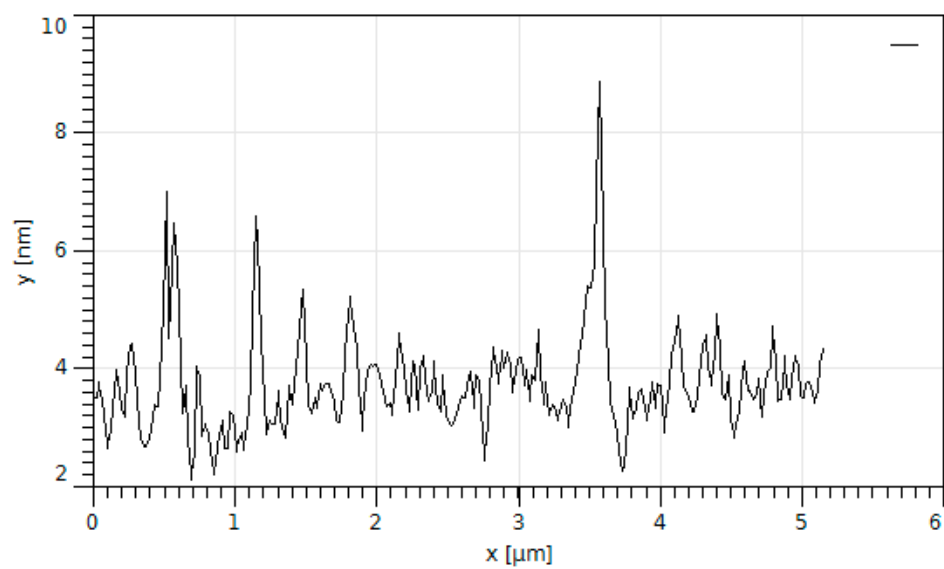

C1

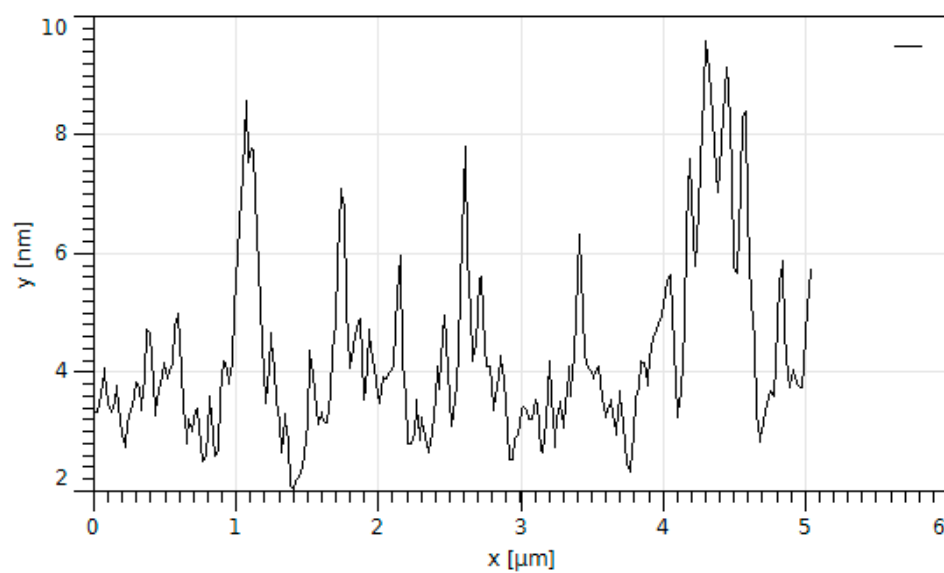

C2

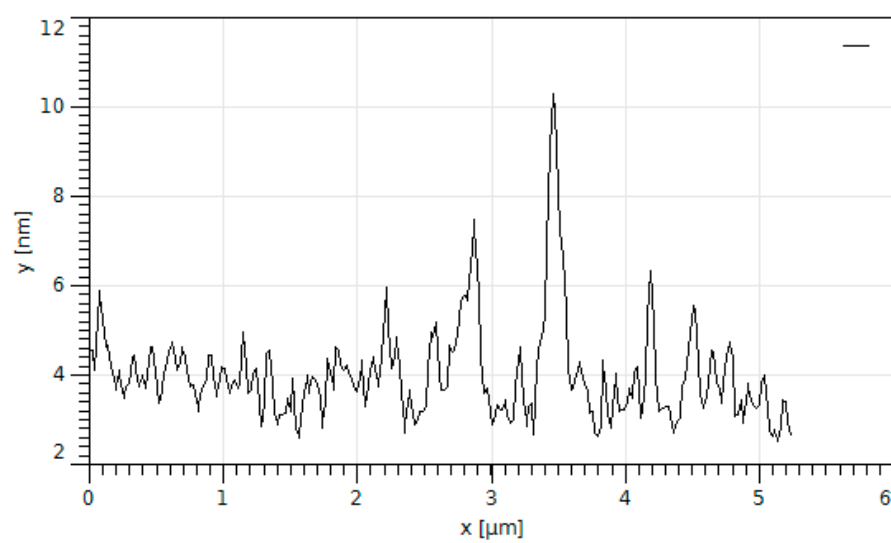

C3

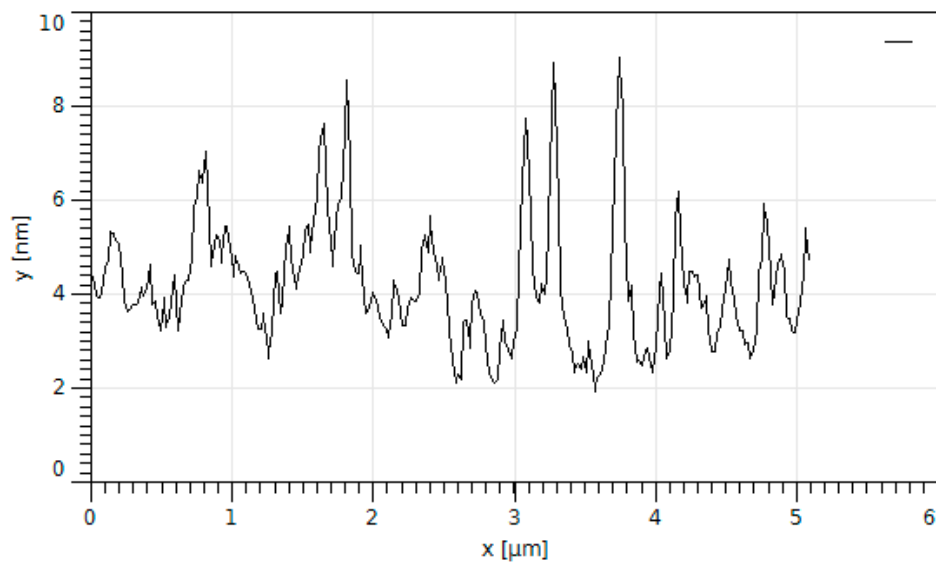

*C4*

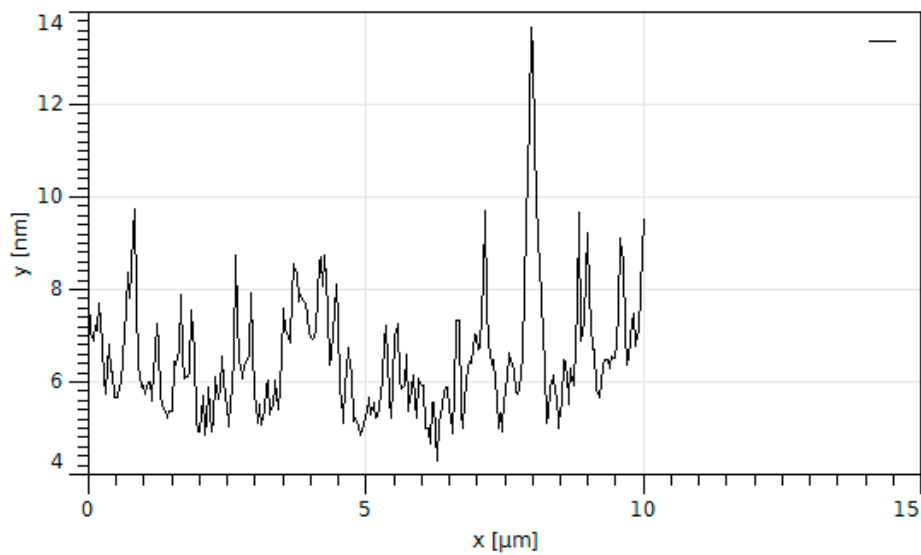

*D1*

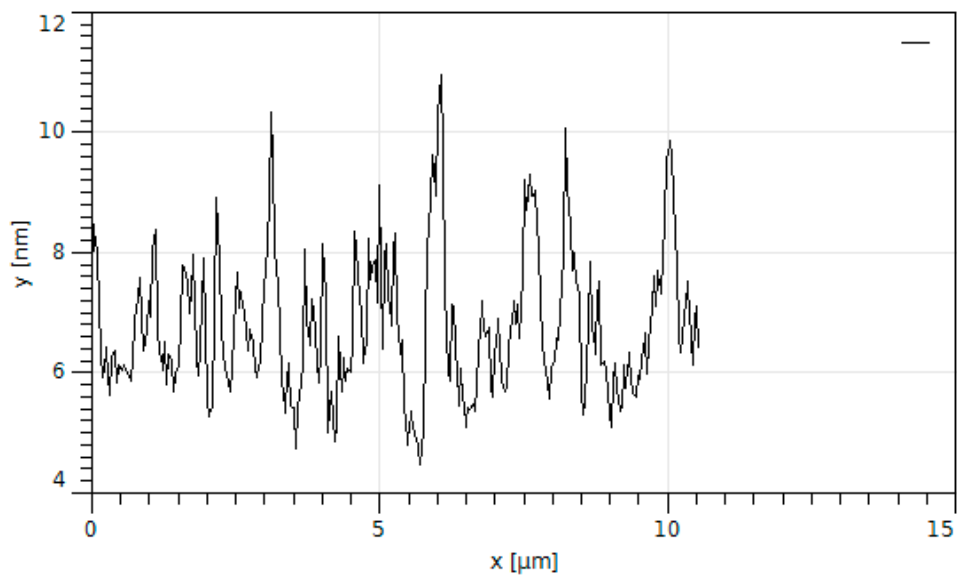

*D2*

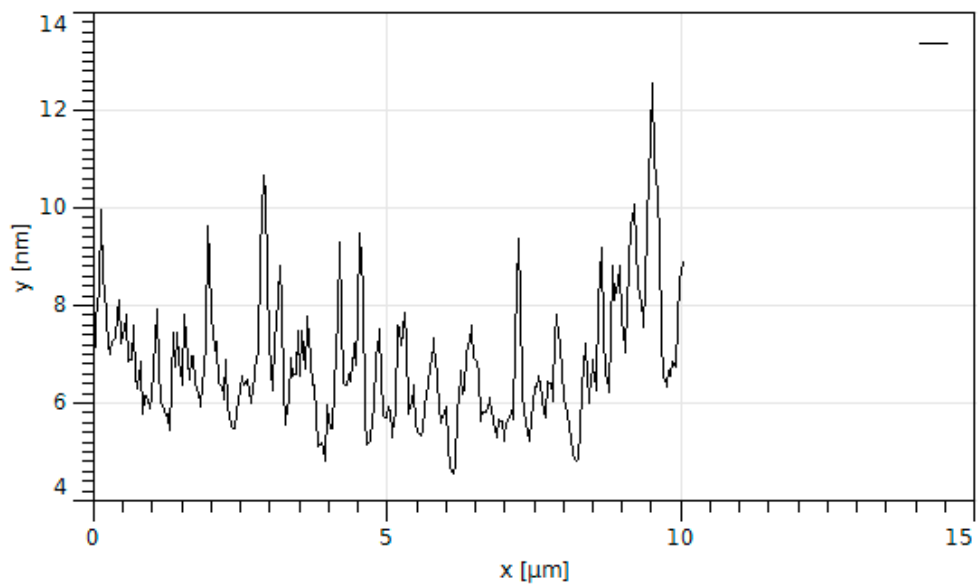

*D3*

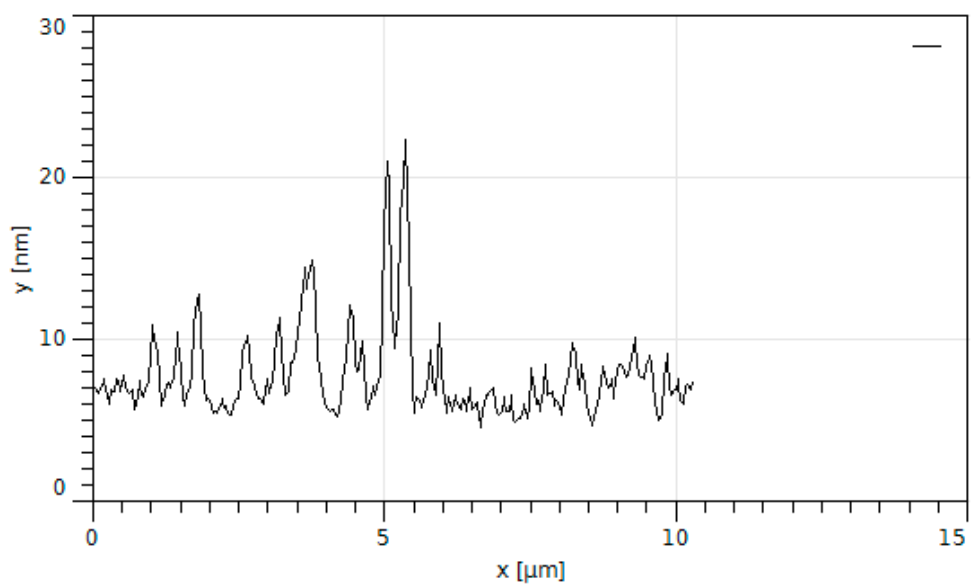

*D4*

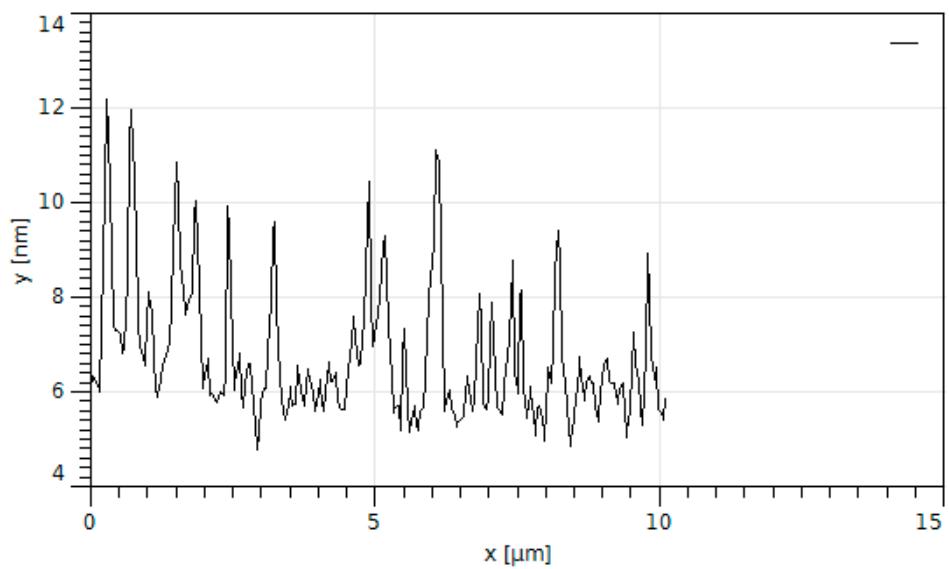

*D5*

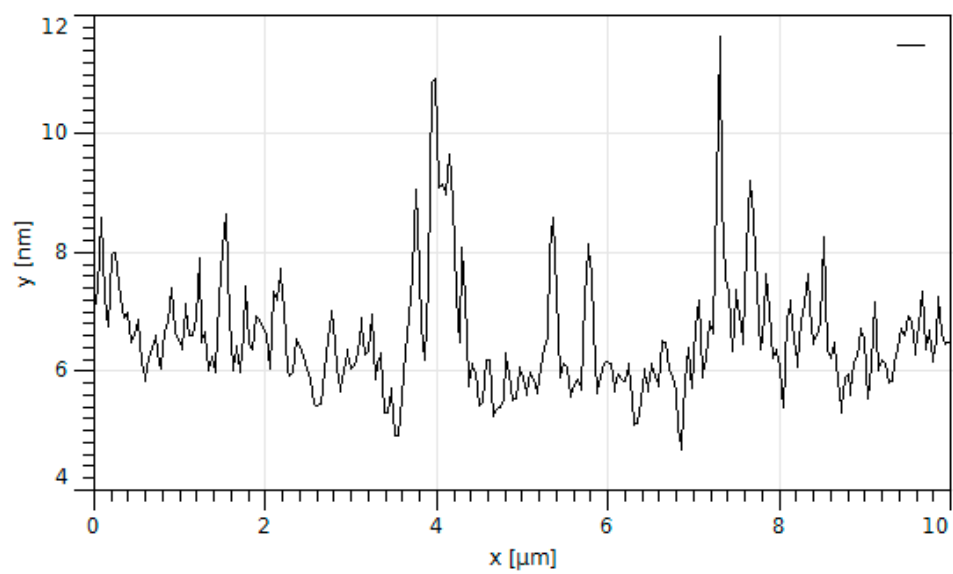

*D6*

Supplement: Supplementary file 1 [file ijms-27-03553-s001.zip › Supplementary File S1.pdf]
